# Species identification of European forest pathogens of the genus *Milesina* (Pucciniales) using urediniospore morphology and molecular barcoding including *M.woodwardiana* sp. nov.

**DOI:** 10.3897/mycokeys.48.30350

**Published:** 2019-03-05

**Authors:** Ben Bubner, Ramona Buchheit, Frank Friedrich, Volker Kummer, Markus Scholler

**Affiliations:** 1 Thünen Institute of Forest Genetics, Eberswalder Chaussee 3a, 15377 Waldsieversdorf, Germany State Museum of Natural History Karlsruhe Karlsruhe Germany; 2 State Museum of Natural History Karlsruhe, Erbprinzenstraße 13, 76133 Karlsruhe, Germany Thünen Institute of Forest Genetics Waldsieversdorf Germany; 3 Karlsruhe Institute of Technology, Competence Center for Material Moisture, Hermann-von-Helmholtz-Platz 1, 76344 Eggenstein-Leopoldshafen Competence Center for Material Moisture Eggenstein-Leopoldshafen Germany; 4 University of Potsdam, Biodiversity Research/Plant Systematics, Maulbeerallee 1, 14469 Potsdam, Germany University of Potsdam Potsdam Germany

**Keywords:** *
Abies alba
*, Polypodiales, GBOL, germ pores, host alternation, *
Uredinopsis
*, Europe

## Abstract

Species of rust fungi of the genus *Milesina* (Pucciniastraceae, Pucciniales) are distributed mainly in northern temperate regions. They host-alternate between needles of fir (*Abies* spp.) and fronds of ferns (species of Polypodiales). *Milesina* species are distinguished based on host taxonomy and urediniospore morphology. In this study, 12 species of *Milesina* from Europe were revised. Specimens were examined by light and scanning electron microscopy for urediniospore morphology with a focus on visualising germ pores (number, size and position) and echinulation. In addition, barcode loci (ITS, nad6, 28S) were used for species delimitation and for molecular phylogenetic analyses. Barcodes of 72 *Milesina* specimens were provided, including 11 of the 12 species.

Whereas urediniospore morphology features were sufficient to distinguish all 12 *Milesina* species except for 2 (*M.blechni* and *M.kriegeriana*), ITS sequences separated only 4 of 11 species. Sequencing with 28S and nad6 did not improve species resolution. Phylogenetic analysis, however, revealed four phylogenetic groups within *Milesina* that also correlate with specific urediniospore characters (germ pore number and position and echinulation). These groups are proposed as new sections within *Milesina* (sections *Milesina*, *Vogesiacae* M. Scholler & Bubner, **sect. nov.**, *Scolopendriorum* M. Scholler & Bubner, **sect. nov.** and *Carpaticae* M. Scholler & Bubner, **sect. nov.**). In addition, *Milesinawoodwardiana* Buchheit & M. Scholler, **sp. nov.** on *Woodwardiaradicans*, a member of the type section Milesina, is newly described. An identification key for European *Milesina* species, based on urediniospore features, is provided.

## Introduction

Several genera of rust fungi (Pucciniales) in Europe alternate their hosts between *Abies* spp. (aecial host with spore states 0 and I) and ferns of the order Polypodiales (telial host with spore states II, III and IV or III and IV). These are species of the genera *Calyptospora* J.G. Kühn (*Thekopsora* Magnus p.p.), *Hyalopsora* Magnus, *Milesina* Magnus (= *Milesia* F.B. White; see Aime et al. 2018b) and possibly *Uredinopsis* Magnus (Gäumann 1959; Klenke and Scholler 2015). All are assigned to the Pucciniastraceae.

The genus *Milesina* Magnus was monographed by Faull (1932). Today, 36 species are known worldwide (Kirk et al. 2001) and 11 species in Europe (Gäumann 1959; Klenke and Scholler 2015). Most species occur in parts of the northern hemisphere with temperate climates and *Abies* populations (Faull 1932). A few species are also found in the southern hemisphere outside the natural area of *Abies* species (Liu 1971), for example, South and Central America, South Africa and New Zealand (Berndt 2008). According to Sinclair and Lyon (2005), species of the genus *Milesina* and *Uredinopsis* (both called fir-fern rust) may cause needle browning and/or defoliation and occasionally cause economic damage in Christmas tree plantations in Canada. Aeciospores from needles infect ferns. Symptoms on fern fronds are pale green to yellow spots, which later become necrotic and are typically confined by veins (Sinclair and Lyon 2005).

Fern rust species on the telial hosts are characterised and distinguished mainly by host taxonomy (telial host genus), size, shape and ornamentation of urediniospores (e.g. Moss 1926, Faull 1932, Berndt 2008) and the results of inoculation experiments (Hunter 1935, 1936, Kamei 1940, Klebahn 1916; Mayor 1944). In contrast to urediniospores, teliospores are either not formed regularly or at all and teliospore features are obscure. So far, it is not possible to morphologically distinguish species on their aecial hosts *Abies* spp. (spore and sori features). Urediniospore features alone were also hardly sufficient to distinguish species (e.g. Faull 1932, Gäumann 1959, Majewski 1977). Thus, fern host identification is often the only criterion to link records to a certain species. This is problematic because some fern species may be infected by two (or possibly even more) *Milesina* spp. (e.g. *Dryopteris* spp. and *Polystichum* spp. in Europe; Gäumann 1959, Klenke and Scholler 2015).

In the present study, the urediniospore morphology of European *Milesina* species was investigated by light and scanning electron microscopical techniques. The morphological approach is supplemented by a molecular phylogenetic approach based on the ITS (Internal Transcribed Spacer) region of the rDNA, which has been shown to be the best marker for barcode species within fungi (Schoch et al. 2012). As secondary barcodes, nad6 (subunit 6 of NADH dehydrogenase) and 28S rDNA have been used. The molecular data were generated within the German Barcode of Life Project GBOL (Geiger et al. 2016). The present study has three objectives, to:

i) provide a detailed morphological description of urediniospores of all European *Milesina* spp., including the development of a method to visualise their germ pores. Germ pores are known to be a valuable taxonomic feature, for example, in grass rust fungi (Cummins 1971). So far, germ pores have not been visualised in the major studies on *Milesina* spp. (Berndt 2008; Faull 1932).

ii) provide molecular barcodes (ITS, nad6, 28S) for Central European species of *Milesina* spp. within the German Barcode of Life project (Geiger et al. 2016).

iii) assess the assignment of morphological species by comparison with the molecular data.

## Methods

### Herbaria

Dried herbarium specimens from the following public herbaria were used: B, FH, G, GLM, GZU, HBG, KR, M, PUR, S and W (acronyms according to Index Herbariorum, Holmgren et al. 1990).

### Light microscopy (LM)

Urediniospores and cross sections of sori (uredinia) from dried *Milesina* specimens were mounted in a mixture of lactic acid and glycerol (Kirk et al. 2001) and examined with a light microscope (Zeiss Axioskop 2 plus) at a magnification of 400× or 1000×. If a sufficient amount of spore material were available, 30 spores per specimen were arbitrarily selected and measured. The number of examined specimens was between two (*M.magnusiana*) and 23 (*M.kriegeriana*). The number of spores examined depended on sample size and varied for each measurement and also between specimens. The length and width of 30 spores (2–4 specimens per species) and sori (only specimens with *Woodwardia* host), the length of 15 spines, the cell wall thickness of 10 spores and the distance between 20 spines were measured for each specimen (2–16 specimens per species). For spine base diameters, see next chapter.

Germ pore number and their position in the wall of urediniospores were evaluated by an adapted technique originally developed for the genus *Tranzschelia* (Scholler et al. 2014). Spores were mounted in Hoyer’s medium (Cunningham 1972) on a slide, then cover slips were pressed until the spores were disrupted and released the plasma. Then the slides were placed on a drier at 40 °C. After two to five days, the numbers of germ pores were counted in phase contrast illumination at 400× magnification for 120 spores of each species. Only the specimens with the best observable germ pores were used for the analysis. In addition, the diameter of pores was measured at 400× magnification.

Specimens were photographed with a Jenoptik ProgRes CT3 digital camera attached to a Zeiss Axioskop 2 plus light microscope (Oberkochen), using differential interference contrast (DIC) and phase contrast as illumination techniques. Images were captured with PROGRES CAPTUREPRO version 2.10.0.1 software. The pictures of the uredinia of *Milesina* sp. were taken with a ProgRes CT3 digital camera (Jena) attached to a Zeiss Stemi 508 (Zeiss, Oberkochen). All values determined in this study were rounded to one decimal place and outliers were not included in the species description.

### Scanning Electron Microscopy (SEM)

Uredinia and urediniospores of dried specimens of *Milesina* spp. were placed on a holder with conductive double-sided tape (Leit-Tabs, Plano GmbH). Scanning electron microscope images were obtained on a Philips XL 30 FEG environmental scanning electron microscope operated at acceleration voltages of 12 kV at a chamber pressure of 133 Pa (1 Torr). In order to achieve a better contrast and less charge effects, the samples were coated first with a mixture of gold (80%) and palladium (20%) (MED 020, BAL-TEC).

SEM studies were carried out to study surface structures which are not visible by light microscopy. Spine base diameters (30 per species) were also measured with SEM and the software IMAGEJ 1.5.

### Statistical Analysis

The statistical analyses for germ pore numbers and boxplots were carried out with the programme R 3.4.3 (R Core Team 2017).

### DNA extraction, PCR and sequencing

Samples were prepared from herbarium specimens by excising single rust pustules including the plant material. They were placed into micro tubes with 8–12 ceramic beads, 1.4 mm diameter (Bio-Budget technologies, Krefeld, Germany), frozen at -20 °C overnight and homogenised on a Bead Ruptor (biolabproducts, Bebensee, Germany) at a speed of 7.45 m/s for 25 s. After freezing the samples again for 10 min at -20 °C, homogenisation was repeated. DNA was extracted with the DNeasy Plant Mini Kit (Qiagen, Hilden, Germany) following the manufacturer’s protocol. Selected samples were homogenised with glass mini mortars and pestles (Roth, Karlsruhe, Germany) in 400 µl of the homogenisation buffer included in the extraction kit.

Molecular barcodes were generated for three loci: ITS (Internal Transcribed Spacer of the ribosomal DNA in the nucleus), 28S (coding for the large subunit of the ribosomal RNA gene located on the ribosomal DNA in the nucleus), nad6 (coding for subunit 6 of NADH dehydrogenase, mitochondrial DNA). Primer sequences are listed in Table 1.

PCR was performed with the Accuprime Taq Polymerase System (Life Technologies, Karlsruhe, Germany) using the supplied buffer II and the following final concentrations: 2 mM MgCl_2_, 0.2 mM of each dNTP and 500 nM of each primer. The PCR programme was as follows: 3 min denaturation at 94 °C, 40 amplification cycles (94 °C for 30 s, 50 °C for 30 s and 68 °C for 60 s) and 7 min strand completion at 68 °C. PCR products were visualised in 1.6% agarose gel. Deviations from the 50 °C annealing temperature are listed in Table 1.

After purification of the PCR product with QIAquick-PCR Purification Kit (Qiagen, Hilden, Germany), it was sent to GATC Biotech AG (Konstanz, Germany) for sequencing. Sequencing was performed with the same primers used for the PCR. Forward and reverse sequences were edited and assembled with the software package GENEIOUS 10.0 (Biomatters, Auckland, New Zealand).

**Table 1.** Primers and PCR conditions.

Locus		Primer	Sequence	Reference	Annealing temperature	Cycle number
ITS	amplicon 1	ITS1F	CTTGGTCATTTAGAGGAAGTAA	(Gardes and Bruns 1993)	60–50 °C	10 cycles with -1 °C per cycle (60–50 °C), then 30 cycles (50 °C)
		ITS4rust	CAGATTACAAATTTGGGCT	(Beenken et al. 2012)		
	amplicon 2*	ITS5u	CAAGGTTTCTGTAGGTG	(Pfunder et al. 2001)	60–50 °C	10 cycles with -1 °C per cycle (60–50 °C), then 30 cycles (60 °C)
		ITS4	TCCTCCGCTTATTGATATGC	(O´Donnell 1993)		
28S	amplicon 1	ITS4BRF	GGACCATGTACAAGTCTGTTGA	(Vialle et al. 2009)	50 °C	40
		LR5	ATCCTGAGGGAAACTTC	(Vilgalys and Hester 2009)		
nad6	amplicon 1	Nad6PucciF1	TTCGATAATAAGTAGCCTAATAGTG	(Vialle et al. 2013)	47 °C	40
		Nad6PucciR1	AAATACAATAGGGCCAATCAT	(Vialle et al. 2013)		

*voucher KR-M-0035533, KR-M-0048135

### Phylogenetic analysis

Several comparison sequences were selected in order to compare the branch length between *Milesina* species with branch lengths between related genera. Criteria of selection were availability within the GBOL project and membership in the Pucciniales suborder Melampsorineae sensu Aime (2006) and Aime et al. (2018b). The genera included *Puccinia* (GenBank) *Pucciniastrum*, *Uredinopsis*, *Cronartium* (GenBank) and *Melampsoridium* as outgroup. GenBank accessions of *Cronartiumribicola* ITS sequences are DQ445908 (Hietala et al. 2008), GU727730 (Mulvey and Hansen 2011) and KX574673 (Vogler et al. 2017). GenBank accessions for *Pucciniagraminis* are AY874141, AY874143 and AY874146 (Abbasi et al. 2005).

Sequences were aligned with the ClustalW algorithm implemented in the programme BioEdit, version 7.1.3.0 (Hall 1999) using the standard parameters offered by the programme. Alignments were used for phylogenetic reconstruction by three different methods:

i) Neighbour-Joining (NJ) analysis was performed with the programme PAUP* 4.0b10 (Sinauer, Sunderland, MA, USA) using the Kimura-2-parameter substitution model. Node support values for NJ were calculated from 1000 bootstrap replicates.

ii) Maximum-Likelihood (ML) analysis: The original NEXUS alignment was reformatted to the extended PHYLIP format using the programme Mesquite 2.75 (http://mesquiteproject.org/mesquite/mesquite.html). The PHYLIP alignment was analysed under the ML criterion on the web-based RAxML black box (Stamatakis et al. 2008https://www.genome.jp/tools/raxml/. Both formats were accessed on 01.09.2018). The used substitution model was GTR without GAMMA correction for amongst-site heterogeneity. Node support values were calculated from 100 bootstrap replicates.

c) Bayesian Inference (BI) analysis: The DNA-Substitution model GTR+I+G was used for performing Bayesian analysis with the programme MrBayes 3.2 (Ronquist et al. 2012). Two independent MCMC runs were performed, each with four chains over 1 000 000 generations. Every 100^th^ tree was sampled. Initial burn-in was 25% and summarisations were calculated after the standard deviation of split frequencies reached below 0.01. The resulting tree file contained posterior probability values for node support.

Tree files resulting from the three methods were visualised using the programme TreeGraph 2 (Stöver and Müller 2010). Alignments are provided as NEXUS files in the Online Supplemental Material (Suppl. materials 1–3).

## Results

### Barcoding success for ITS, nad6 and 28S

ITS sequences were generated for 72 specimens of 11 *Milesina* species (Table 2). These include 10 of 11 *Milesina* species known to be present in Europe. Only for *M.magnusiana* no material was available. In addition, we sequenced an unknown *Milesina* species from the Canary Islands which politically belongs to Europe but geographically may belong to Africa. All specimens are from the fungus collection of the State Museum of Natural History Museum Karlsruhe, Germany (Acronym KR) as listed in the Methods section. An additional 43 specimens (data not shown) yielded no sequences. Thus, the success rate for sequencing was 63%. Amongst the unsuccessful specimens, 17 were collected before 2010 and 12 failed specimens were collected in 2017. The oldest successfully sequenced specimens were from 1999 (*M.murariae*, KR-M-0025191; *M.scolopendrii* KR-M-0025400). No attempts have been made to sequence *M.magnusiana* because only two old species from 1933 and 1964 (M-0290299, M-0205474) were available. Nine ITS sequences were generated for the genera *Chrysomyxa*, *Melampsoridium* and *Uredinopsis* (Table 3).

**Table 2.** *Milesina* specimens: herbarium, lab and GenBank accession numbers.

Species	Host plant species	Voucher (all herbarium KR)	Lab no.	ITS	28S	nad6
* M. blechni *	* Struthiopteris spicant *	KR-M-0038517	B1426	MH908410		
	* Struthiopteris spicant *	KR-M-0038523	B1427	MH908411		
	* Struthiopteris spicant *	KR-M-0038519	B1428	MH908412	MK302189	
	* Struthiopteris spicant *	KR-M-0038516	B1442	MH908421	MK302193	
	* Struthiopteris spicant *	KR-M-0049039	B1893	MH908463		
* M. carpatica *	* Dryopteris filix-mas *	KR-M-0048589	B1662	MH908451		
	* Dryopteris filix-mas *	KR-M-0043192	B1780	MH908454		
* M. exigua *	* Polystichum braunii *	KR-M-0050247	B2206	MH908478	MK302211	MK302182
* M. feurichii *	* Asplenium septentrionale *	KR-M-0043159	B1964	MH908476		
* M. kriegeriana *	* Dryopteris carthusiana *	KR-M-0043170	B1435	MH908417		
	* Dryopteris dilatata *	KR-M-0043182	B1438	MH908418		
	* Dryopteris dilatata *	KR-M-0043165	B1440	MH908419	MK302191	
	* Dryopteris dilatata *	KR-M-0039321	B1441	MH908420	MK302192	
	* Dryopteris carthusiana *	KR-M-0048087	B1469	MH908441	MK302203	
	* Dryopteris carthusiana *	KR-M-0048085	B1470	MH908442	MK302204	MK302166
	* Dryopteris carthusiana *	KR-M-0048086	B1471	MH908443	MK302205	
	* Dryopteris dilatata *	KR-M-0043162	B1472	MH908444		
	* Dryopteris dilatata *	KR-M-0048088	B1473	MH908445		
	* Dryopteris dilatata *	KR-M-0043151	B1474	MH908446		
	* Dryopteris dilatata *	KR-M-0043184	B1475	MH908447		
	* Dryopteris filix-mas *	KR-M-0043178	B1476	MH908448		
	* Dryopteris dilatata *	KR-M-0048357	B1494	MH908449		
	* Dryopteris dilatata *	KR-M-0048477	B1602	MH908450	MK302206	
	* Dryopteris dilatata *	KR-M-0048480	B1685	MH908452	MK302207	
* M. murariae *	* Asplenium ruta-muraria *	KR-M-0048133	B1443	MH908422	MK302194	
	* Asplenium ruta-muraria *	KR-M-0048134	B1444	MH908423	MK302195	
	* Asplenium ruta-muraria *	KR-M-0048132	B1445	MH908424	MK302196	
	* Asplenium ruta-muraria *	KR-M-0035461	B1446	MH908425	MK302197	MK302150
	* Asplenium ruta-muraria *	KR-M-0036224	B1447	MH908426		MK302151
	* Asplenium ruta-muraria *	KR-M-0036225	B1448	MH908427		MK302152
	* Asplenium ruta-muraria *	KR-M-0025768	B1449	MH908428		MK302153
	* Asplenium ruta-muraria *	KR-M-0025185	B1450	MH908429		MK302154
	* Asplenium ruta-muraria *	KR-M-0025184	B1451	MH908430		MK302155
	* Asplenium ruta-muraria *	KR-M-0025191	B1452	MH908431		MK302156
	* Asplenium ruta-muraria *	KR-M-0043149	B1852	MH908459		MK302168
	* Asplenium ruta-muraria *	KR-M-0043154	B1853	MH908460		MK302169
* M. polypodii *	* Polypodium vulgare *	KR-M-0043177	B1429	MH908413		
	* Polypodium interjectum *	KR-M-0043189	B1431	MH908414		
	* Polypodium vulgare *	KR-M-0043190	B1432	MH908415	MK302190	
	* Polypodium vulgare *	KR-M-0043161	B1433	MH908416		
	* Polypodium vulgare *	KR-M-0043152	B1466	MH908439		MK302164
	* Polypodium vulgare *	KR-M-0048818	B1846	MH908455		
	* Polypodium vulgare *	KR-M-0043157	B1847	MH908456		
	* Polypodium vulgare *	KR-M-0043146	B1848	MH908457		
	* Polypodium vulgare *	KR-M-0043173	B1849	MH908458		
	* Polypodium vulgare *	KR-M-0048694	B1778	MH908453		MK302167
* M. scolopendrii *	* Asplenium scolopendrium *	KR-M-0043186	B1455	MH908434	MK302198	MK302159
	* Asplenium scolopendrium *	KR-M-0043153	B1456	MH908435		MK302160
	* Asplenium scolopendrium *	KR-M-0025400	B1457	MH908436	MK302199	MK302161
	* Asplenium scolopendrium *	KR-M-0049066	B1896	MH908464		MK302170
	* Asplenium scolopendrium *	KR-M-0049049	B1897	MH908465		MK302171
	* Asplenium scolopendrium *	KR-M-0049050	B1898	MH908466	MK302208	MK302172
	* Asplenium scolopendrium *	KR-M-0049051	B1899	MH908467	MK302209	MK302173
*M.* sp.	* Abies alba *	KR-M-0043687	B1458	MH908437	MK302200	MK302162
	* Abies alba *	KR-M-0042052	B1459	MH908438	MK302201	MK302163
	* Abies alba *	KR-M-0018587	B1860	MH908461		
	* Abies alba *	KR-M-0018624	B1861	MH908462		
	* Abies alba *	KR-M-0049062	B1902	MH908468		
	* Abies alba *	KR-M-0049038	B1903	MH908469		
	* Abies alba *	KR-M-0049065	B1905	MH908470		MK302174
	* Abies alba *	KR-M-0049068	B1906	MH908471		MK302175
	* Abies alba *	KR-M-0049063	B1907	MH908472		
	* Abies alba *	KR-M-0048773	B1911	MH908473		
	* Abies alba *	KR-M-0050303	B2209	MH908480	MK302215	MK302184
* M. vogesiaca *	* Polystichum aculeatum *	KR-M-0003937	GBOL_1_f10	MH908490		
	* Polystichum aculeatum *	KR-M-0043175	B1453	MH908432		MK302157
	* Polystichum aculeatum *	KR-M-0043160	B1454	MH908433		MK302158
	* Polystichum aculeatum *	KR-M-0043187	B1467	MH908440	MK302202	MK302165
* M. whitei *	* Polystichum aculeatum *	KR-M-0049177	B1965	MH908477		
	* Polystichum aculeatum *	KR-M-0050248	B2207	MH908479	MK302212	
***M.woodwardiana* sp. nov.**	* Woodwardia radicans *	KR-M-0049033	B1912	MH908474		MK302176
	* Woodwardia radicans *	KR-M-0048787	B1914	MH908475		

All 72 specimens with ITS sequences were sequenced for the loci nad6 and 28S. Twenty nine specimens yielded barcode sequences at the locus nad6 (sequencing success 40%), while 24 specimens were successfully sequenced at the locus 28S (sequencing success 33%, Table 2). Since no nad6 or 28S sequences could be generated for specimens of the genus *Chrysomyxa*, specimens of the genus *Pucciniastrum* were sequenced as replacements in nad6 and 28S phylograms (three sequences for nad6, one sequence for 28S, Table 3).

**Table 3.** ITS barcodes specimens of Pucciniastraceae other than *Milesina*: herbarium, lab and accession numbers.

Species	host plant species	voucher (all herbarium KR)	lab no.	ITS	28S	nad6
* Chrysomyxa empetri *	* Empetrum hermaphroditum *	KR-M-0040758	B1252	MH908481		
* Chrysomyxa pyrolata *	* Pyrola minor *	KR-M-0048660	B1688	MH908484		
	* Pyrola rotundifolia *	KR-M-0048741	B1689	MH908485		
* Melampsoridium betulinum *	* Betula pendula *	KR-M-0035533	B1412	MH908482	MK302186	
	* Betula pubescens *	KR-M-0048135	B1416	MH908483	MK302187	
	* Betula pubescens *	KR-M-0048557	B1835	MH908487		
* Melampsoridium carpini *	* Carpinus betulus *	KR-M-0048587	B1774	MH908486		
* Melampsoridium hiratsukanum *	* Alnus incana *	KR-M-0049100	B2033			MK302178
	* Alnus glutinosa *	KR-M-0048149	B1420		MK302188	
* Pucciniastrum circaeae *	* Circaea intermedia *	KR-M-0039060	B2038			MK302179
* Pucciniastrum epilobii *	* Epilobium ciliatum *	KR-M-0004576	B2039			MK302180
	* Epilobium palustre *	KR-M-0043058	B2040		MK302210	MK302181
* Uredinopsis filicina *	* Phegopteris connectilis *	KR-M-0050249	B2208	MH908488	MK302213	MK302183
	* Phegopteris connectilis *	KR-M-0012195	B2011			MK302177
	* Phegopteris connectilis *	KR-M-0050313	B2212	MH908489	MK302215	MK302185

### Phylogenetic analysis of the ITS barcode

**Figure 1.**
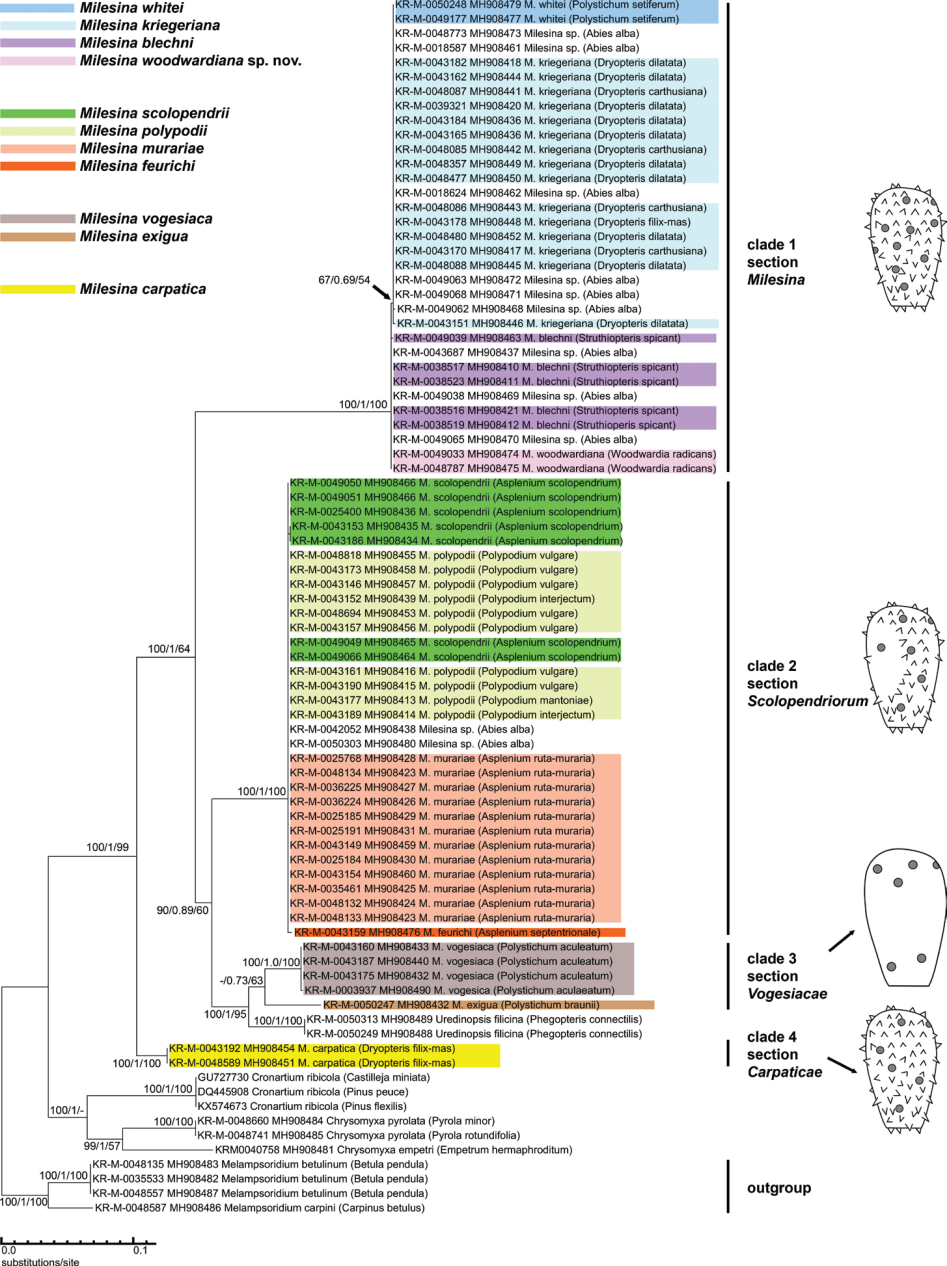
ITS Phylogram of *11 Milesina species* (excluding *M.magnusiana*). The phylogram is based on a 733-bp alignment. A Maximum Likelihood (ML) tree is shown with support values for ML, Bayesian Inference (BI) and Neighbour Joining (NJ), in the order ML/BI/NJ. Support values are presented when they are above 50 (ML, NJ) or 0.5 (BI). The host is indicated in brackets. *Milesina* specimens without species designation (host *Abiesalba*) are not colour-coded. For comparison, several sequences were included from closely related genera. They were all newly generated within the GBOL project, except the GenBank sequences for *Cronartium* spp. The drawings on the right side present the typical arrangement of spines and germ pores (grey dots) on the *Milesina* urediniospores.

Phylogenetic analysis of the ITS barcode revealed four clades for clades within *Milesina* species. The nodes for the first, second and fourth clade have maximum support values of 100/1/100 for the three phylogenetic reconstruction methods ML, BI and NJ (Figure 1). In clade 1, the ITS sequences of the pairs *M.whitei*/*kriegeriana* and *M.blechni*/*woodwardiana* sp. nov. are almost identical within the pairs but differ between the pairs by one nucleotide (Figure 3). At position 381, the nucleotide T (*M.whiteikriegeriana*) is replaced by C (*M.blechni*/*M.woodwardiana* sp. nov.). Position 1 is the first nucleotide after the signature TCATTA for the 3´end of the 18S rDNA. This difference is also reflected in the ITS phylogram by a node with weak support of 67/0.69/54 (Figure 1).

In clade 2, *M.scolopendrii*, *M.polypodii* and *M.murariae* cannot be distinguished by ITS sequences. Apart from single nucleotides at unspecific positions, the ITS sequences are identical. The sequence of *M.feurichii* differs from the other three species by two nucleotides with specific positions (Figure 4). This difference, however, is not reflected by bootstrap or posterior probability support (Figure 1). Clade 3 with *M.vogesiaca* and *M.exigua* has only weak support in the BI and NJ analysis (0.73 and 63) while the bootstrap support was below 50 for the ML analysis (Figure 1). In a version of the ITS phylogram with *Pucciniagraminis* as outgroup (Online Suppl. material 1: Figure S1), the support values for clade 3, including *M.exigua* are 100/1/100. The only member of the fourth clade is *M.carpatica* with two identical sequences. The ITS sequence is clearly different from all other *Milesina* species investigated. In summary, amongst the 11 *Milesina* species, only four species (*M.feurichii*, *M.vogesiaca*, *M.exigua* and *M.carpatica*) can be unambiguously assigned by their ITS sequences.

Amongst the specimens on the aecial host *Abiesalba*, nine grouped into clade 1 and two into clade 2 (Figure 1). Following the distinction at position 381, six *Abies*-dwelling specimens of clade 1 belong to the pair *M.whitei*/*kriegeriana* and three to the pair *M.blechni*/*woodwardiana* sp. nov.

The ITS phylogeny (Figure 1) does not confirm monophyly of *Milesina* species. High support values are available only for a clade containing all *Milesina* species and *Uredinopsisfilicina* (node support (100/1/99, Figure 1). This indicates that the genus *Milesina* may be paraphyletic. The specimens of the three genera *Melampsoridium*, *Cronartium* and *Chrysomyxa* form a clade with a node support of 81/-/0.9, indicating that probably none of them is a sister group of *Milesina*. When the branch lengths from the clade defining node to the next deeper node are compared, it its apparent that the branch lengths for the *Milesina* clades are as long or even longer when compared to the branch lengths between the different genera *Melampsoridium*, *Cronartium* and *Chrysomyxa*. This indicates a relatively large genetic distance between the clades within the genus *Milesina*.

### Phylogenetic analysis of nad6 and 28S barcodes

**Figure 2.**
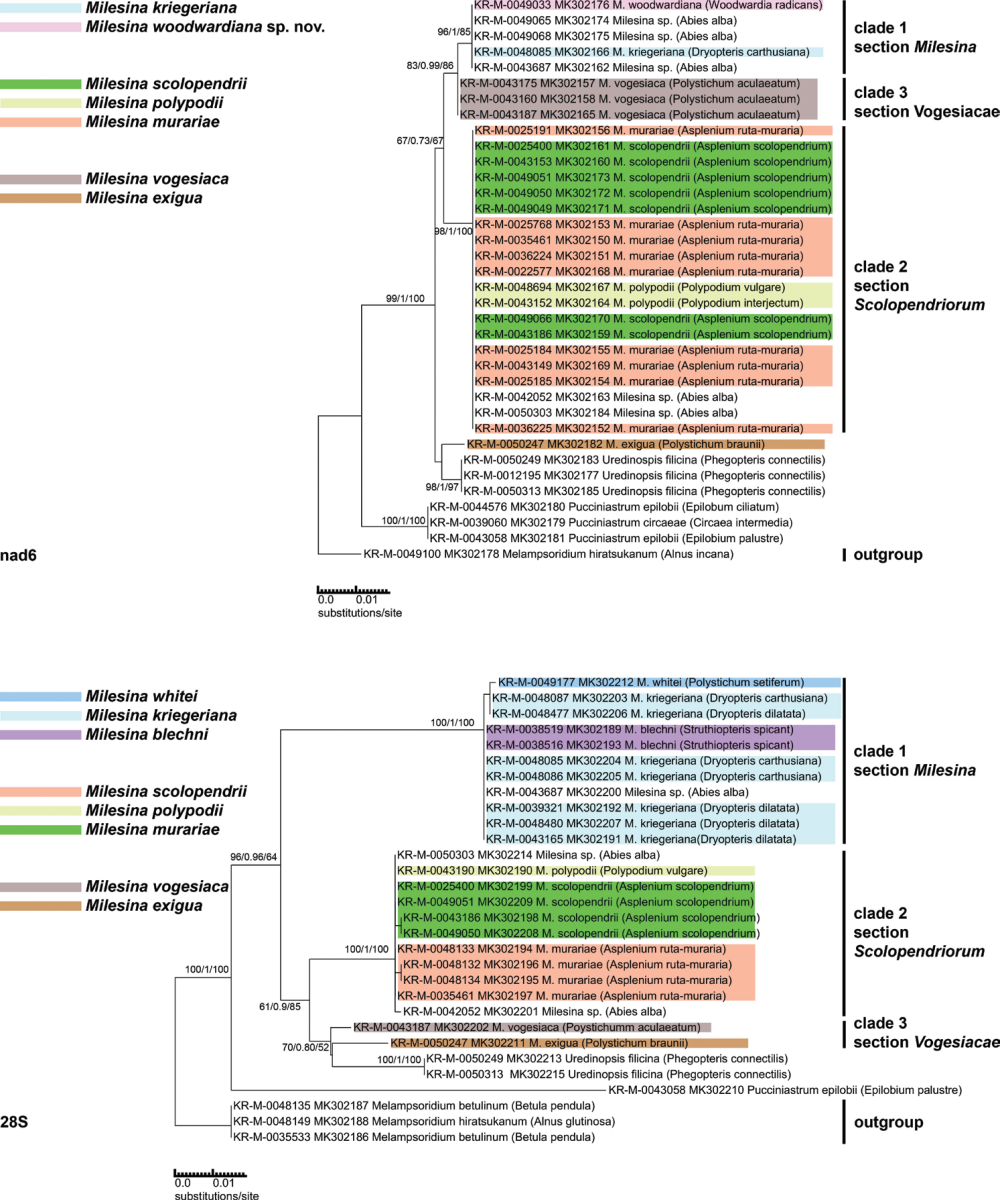
Phylograms of supplementary barcodes. The nad6 phylogram is based on a 550 bp alignment, the 28S phylogram on a 680 bp alignment. The technical description is the same as for Figure 1. All *Milesina* specimens from Figure 1 were attempted to sequence for the supplementary barcodes. Only the shown specimens resulted in sequences. The non-*Milesina* species were altered depending on availability. No GenBank sequences were included and the genus *Chrysomyxa* was replaced by *Pucciniastrum*.

Due to the low sequencing success of these two markers, only seven (nad6) and eight (28S) *Milesina* species could be included in the analysis. Although no sequences are available for clade 4 (*M.carpatica*), the general pattern of the clades is the same as for the ITS phylogeny. Clade 1 and clade 2 consist of the same species (Figure 2) as in the ITS analysis and cannot be distinguished. In addition and in contrast to the ITS data, the distinction between the species pairs *M.whitei/kriegeriana* and *M.blechni*/*woodwardiana* sp. nov. is not possible. In confirmation of the ITS data, the support for a clade that contains all *Milesina* species and *Uredinopsisfilicina* is high (99/1/100 for nad6, 96/0.96/64 for 28S, Figure 2). This again indicates that the genus *Milesina* is not monophyletic. The branch lengths from the clade defining node to the next deeper node are shorter between the *Milesina* clades as compared to the branch length between related species. This is in contrast to ITS data.

**Figure 3.**
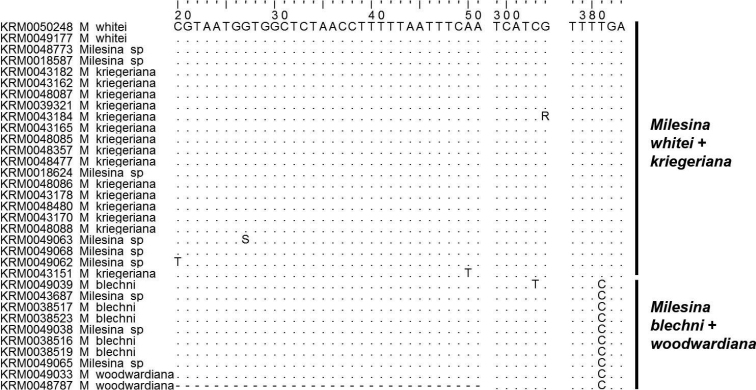
Deviations from the consensus ITS sequence of section Milesina. The first line indicates the nucleotide positions in base pairs, the second line the consensus sequence. The order of specimens is as shown in Figure 1. “*Milesina* sp” denotes specimens from *Abiesalba*. Deviations for single specimens can be found at 5 positions. All specimens of *M.blechni* and *M.woodwardiana* deviate at position 381 from *M.whitei* and *kriegeriana*.

The ambiguity in ITS data to determine a clade 3, consisting of both *M.vogesiaca* and *M.exigua*, is also found in the nad6 and 28S data. In the nad6 phylogram, *M.exigua* has an unsupported position next to *Uredinopsisfilicina*. In the 28S phylogram, *M.exigua* is only in the same clade with *M.vogesiaca* if *Uredinopsisfilicina* is included. Even then, the support values of 70/0.8/52 are relatively low.

### Morphology of urediniospores

Germ pores

The number and position of the germ pores of all species were visualised. Germ pores provided three important features, namely (i) the number, (ii) the position and, finally, (iii) the size of pores. The four species with the highest number of germ pores per spore all belong to the section Milesina (Figure 5). All other species had similar germ pore numbers.

**Figure 4.**
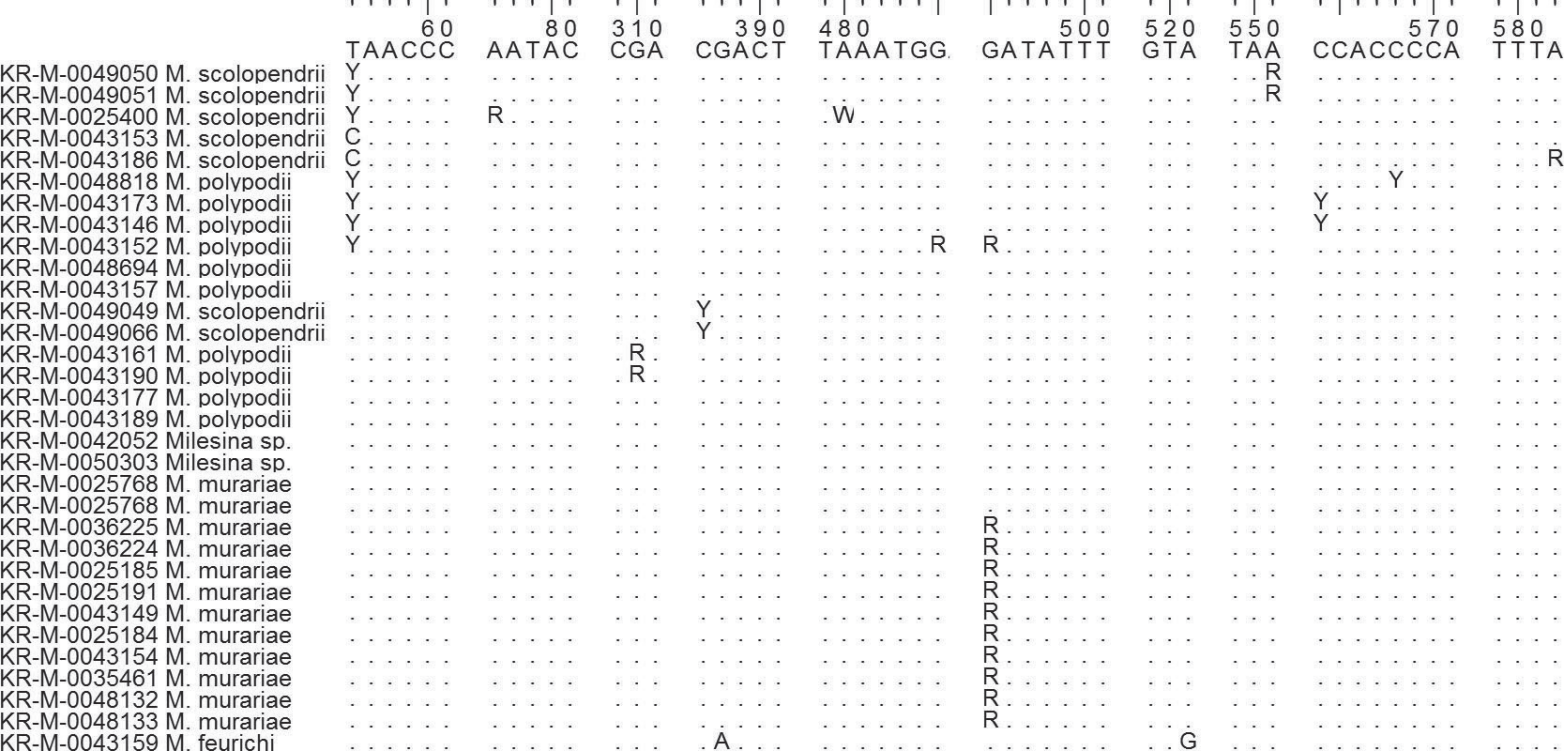
Deviations from the consensus ITS sequence of section Scolopendriorum. Description as for Figure 3. *Milesinafeurichii* deviates from the other three species in positions 288 (A) and 521 (G).

**Figure 5.**
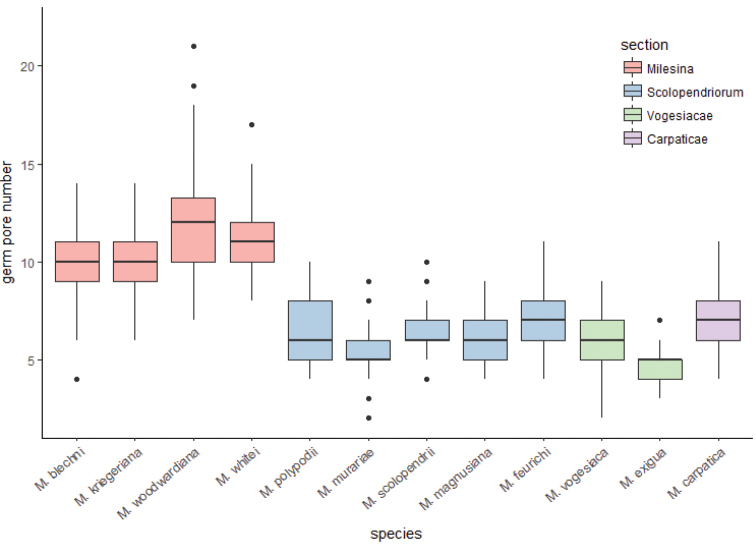
Boxplot of germ pore numbers of urediniospores of 12 *Milesina* spp. and four sections. For each species 120 spores from two (*M.magnusiana*), three (*M.feurichii*) or four (all other species) specimens were evaluated. Median, whisker, quantile and outliers (dots) are shown.

## Specimens examined, urediniospore descriptions and taxonomic novelties

Comparative data of the main morphological spore characters are listed in Table 4.

**Table 4.** Comparative overview of morphological features of urediniospores and host range in *Milesina*.

Species	Host plant genus (family)	Frequent spine length [µm]	Smooth spine-free areas	Frequent wall thickness [µm]	Frequent germ pore number	Ø germ pore diam. [µm]	Germ pore distri-bution	Frequent spore size [µm]	Other
* Milesina blechni *	*Struthiopterisspicant* (Blechnaceae)	1.5–2.0	no	0.8–1.0	10–11	2.4	scattered	30.0–37.5 × 15.0–19.0	distance between spines mostly 1.5–4.0 µm, spines typically perpendicular to the wall
* Milesina carpatica *	*Dryopterisfilix-mas* (Dryopteridaceae)	1.0–1.8	no	0.5–1.2	5–7	2.2	scattered	20.0–30.0 × 12.5–19.0	distance between spines mostly 0.5–3.0 µm, spines typically erect
* Milesina exigua *	*Polystichumaculeatum*, *P.braunii*, (Dryopteridaceae)	no spines	no spines	0.5–0.8	4–6	2.7	bizonate	22.5–30.0 × 12.5–17.5	Germ pores concentrated apically or nearly bizonate
* Milesina feurichii *	*Aspleniumseptentrionale* (Aspleniaceae)	±2.0	yes	0.5–1.0	6–7	2.4	scattered	30.0–37.5 × 20.0–22.5	distance between spines mostly 1.0–5.0 µm, spines typically erect
* Milesina kriegeriana *	*Dryopterisborreri*, *D.carthusiana*, *D.dilatata*, *D.filix-mas* (Dryopteridaceae)	±2.0	no	0.8–1.0	10–11	2.3	scattered	27.5–37.5 × 15.0–20.0	distance between spines mostly 1.0–4.0 µm, spines typically erect
* Milesina magnusiana *	*Aspleniumadiantum-nigrum* (Aspleniaceae)	±2.0–2.2	yes	1.0–1.5	5–6	2.9	scattered	30.0–35.0 × 17.5–20.0	distance between spines mostly 3.0–5.5 µm
* Milesina murariae *	*Aspleniumruta-muraria* (Aspleniaceae)	±2.0	yes	2.0	5–6	2.4	scattered	27.5–35.0 × 17.5–22.5	distance between spines 2.0–3.5 µm, spines typically erect, curved near base
* Milesina polypodii *	*Polypodiuminterjectum*, *P.×mantoniae*, *P.vulgare* (Polypodiaceae)	±2.0	yes	0.5–1.0	5–6	2.3	scattered	30.0–40.0 × 17.5–22.5	distance between spines 1.0–4.0 µm, spines typically erect
* Milesina scolopendrii *	*Aspleniumscolopendrium* (Aspleniaceae)	±2.0	yes	0.5–1.2	6–7	2.4	scattered	27.5-42.5 × 17.5-22.5	distance between spines 2.0-5.0 µm, spines typically erect
* Milesina vogesiaca *	*Polystichumaculeatum, P. lonchites* (Dryopteridaceae)	no spines	no spines	0.5-0.8	5-6	2.8	± bizonate	30.0-40.0 × 17.5-20.0	spores with very inconspicuous flat verrucae (visibly with SEM only)
* Milesina whitei *	*Polystichumaculeatum, P.setiferum* (Dryopteridaceae)	1.8-2.5	no	0.8-1.0	9-13	2.3	scattered	27.5-37.5 × 17.5-22.5	distance between spines mostly around 2.0 µm, spines typically perpendicular to the wall
***Milesinawoodwardiana* sp. nov.**	*Woodwardiaradicans* (Blechnaceae)	±3.0	no	0.5-1.0	10-14	2.4	scattered	30.0-37.5 × 17.5-22.5	distance between spines mostly 2.0-4.0 µm, spines irregularly directed

### Milesina blechni

Taxon classificationFungiPuccinialesPucciniastraceae

(Syd. & P. Syd.) Syd. & P. Syd., Annales Mycologici 8(5): 491 (1910)

Figure 6a, b

Struthiopteris spicant (L.) Weiss (Blechnumspicant (L.) Sm.), Czech Republic, Mähren: Hochgesenke, Großer Kessel (Velká kotlina), 19 Mar 1923, F. Petrak, II (W, 1970-25718); Hochgesenke, Großer Kessel (Velká kotlina), 3 Sep 1923, F. Petrak, II (W, 1992-14461); Denmark: 26 Nov 1926, J. Lind, II (W, 1975-19656); 26 Nov 1926, J. Lind, II (W, 1931-7888); France, Alsace: Frankental, Hohneck, 16 Jul 1910, H. Sydow, II (Sydow, Mycoth. Germ. 877; W, 1910-6976, 1973-30378; S, F310830); Germany, Bayern: Aschau, 25 Aug 1934, E. Eichhorn & H. Poeverlein, II (W, 1975-15534); Dreisessel, 12 Oct 1940, E. Eichhorn, II (Sydow, Mycoth. germ. 3449; W, 1942-2122m 1972-17207); Baden-Württemberg, Schwarzwald, St. Georgen, Aug 1913, P. Sydow (Sydow, Uredineen 2739; GLM, GLM-53029; W, 1916-4273; S, F310826); Schwarzwald, path between Bad Wildbad and Kaltenbronn, 13 Aug 1910, P. Sydow, II (S, F310827); Freudenstadt, Baiersbronn, NSG “Wilder See- Hornisgrinde”, coniferous wood, 1 Mar 2014, M. Scholler, II (KR, KR-M-0038516); Schwarzwald, Ortenau, Durbach, Tiefenspring, Großer Langenbach, 15 Sep 2017, R. Buchheit (KR, KR-M-0049039); Schwarzwald, Ortenau, Seebach, coniferous forest, 1 Mar 2014, M. Scholler (KR, KR-M-0038517); Schwarzwald, Rastatt, Forbach, Herrenwieser See, wayside, 15 May 2014, M. Scholler (KR, KR-M-0038519); Schwarzwald, Rastatt, Forbach, Herrenwies, spruce-fir forest, 15 May 2014, M. Scholler, II (KR, KR-M-0038523); Hamburg: Harburg, Klecker Wald, 12 May 1915, O. Jaap (Jaap, Fungi sel. exs. 774; W, 1916-4246); Niedersachsen: Harz, Rehberger Graben, between Oderteich and St. Andreasberg, 24 Aug 1904, P. Sydow, II (Sydow, Mycoth. Germ. 311; S, F310828; W, 1905-4478); Sachsen: Schmilka, Großer Winterberg, 28 Aug 1903, H. and P. Sydow, II (Sydow, Uredineen 1841; B, B 700016503; S, F29283, F310855; W, 1904-006026, type); Schmilka, 28 Aug 1903, H. & P. Sydow, II (H. & P. Sydow, Mycoth. Germ. 61; W, 1903-0013406; S, F29284, type); Schmilka, Großer Winterberg, 28 Aug 1903, P. Sydow, II (S, F29285, type); Thüringen, Stützerbach near Ilmenau, 20 Jul 1911, O. Jaap, II (W, 1911-7479); Eichsfeld, Fretterode Schierbachtal, spruce forest, 14 Nov 2013, H. Thiel, II (KR, KR-M-0043148); Nordrhein-Westfalen, Lennestadt, Forsthaus Einsiedelei, forest, 5 Sep 1919, A. Ludwig, II (W, 1975-12517); Nordrhein-Westfalen: Lennestadt, Forsthaus Einsiedelei, forest, 31 Aug 1921, A. Ludwig, II (W, 1973-16286); Olpe, Forsthaus Einsiedelei, forest, 31 Aug 1921, A. Ludwig, II (W, 1926-23840); Olpe, Silberg, 12 Aug 1931, A. Ludwig, II (W, 1973-17007); Olpe, Silberg, 6 Sep 1940, A. Ludwig, II (W, 1975-20448); Poland, Riesengebirge (Karkonosze): Karpacz (formerly Krummhübel), 29 Aug 1908, H. Sydow, II (S, F310856); Karpacz (formerly Krummhübel), Hoserweg, 29 Aug 1908, H. Sydow, II (S, F310829); Romania: Brasov, mountain chain Fagaras, Valea Sâmbetei, northern Cabana Valea Sâmbetei, Piceetum, 17 Aug 1983, G. Negrean, II (W, 1997-00694); Switzerland, Berner Oberland: Längenbalm, Hasleberg, 3 Sep 1906, E. Fischer, II (B, B 700016504, W, 1907-17136); Bern, Wälde, northern side of the Schwendelberg, next to Guggisberg, 27 Aug 1923, E. Fischer, II (W, 1971-30213).

#### Description.

Urediniospores hyaline, ellipsoidal to obovoidal, clavate, 27.5–42.5 × 15.0–20.0 µm, mostly 30.0–37.5 × 15.0–19.0 µm; wall 0.5–1.5 µm, mostly 0.8–1.0 µm thick; echinulate without spine-free areas, spines 1.2–2.2 µm, mostly 1.5–2.0 µm long, irregularly distributed, sometimes also in rows, spines typically straight and perpendicular to the wall, distance between spine bases 1.0–5.0 µm, mostly 1.5–4.0 µm, spine base 0.7–1.3 µm, mostly 0.9–1.1 µm diam.; germ pores scattered, 6–13, mostly 10–11, 2.0–3.0 µm diam., Ø 2.4 µm diam.

#### Comment.

Urediniospore features are very similar to those of *M.kriegeriana*. Average urediniospore length measurements are somewhat higher (30.0–37.5 vs. 27.5–35.0 in *M.kriegeriana*).

**Figure 6.**
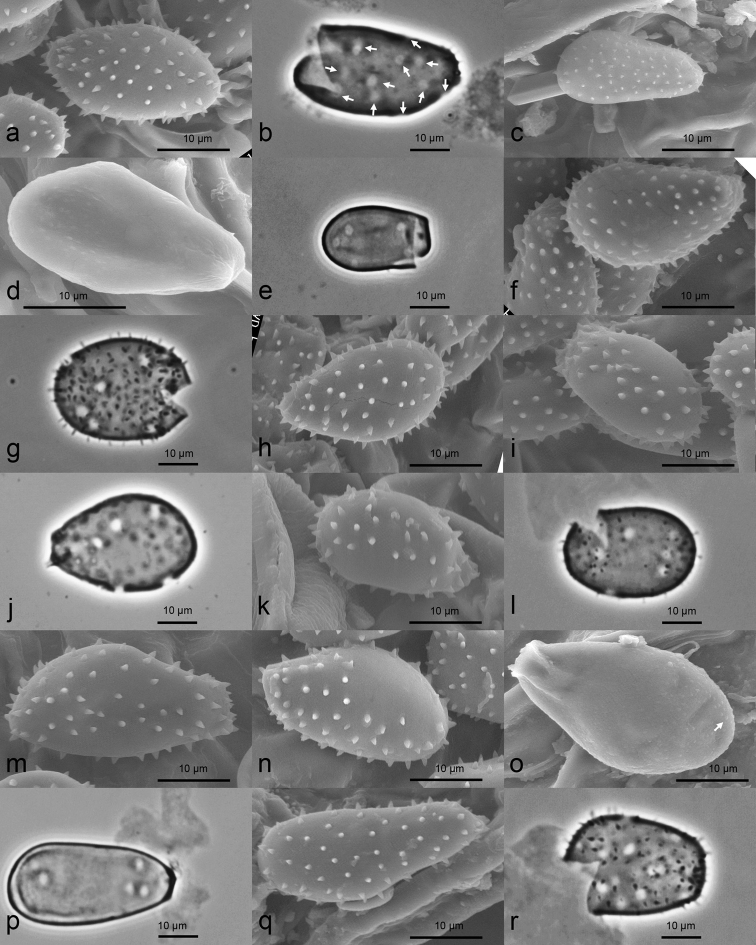
Urediniospores of 11 *Milesina* species. **a***Milesinablechni* on *Struthiopterisspicant* (KR-M-0049039, SEM) **b***Milesinablechni* on *Struthiopterisspicant*, cracked spore with released plasma, germ pores scattered (KR-M-0038523, LM phase contrast) **c***Milesinacarpatica* on *Dryopterisfilix-mas* (KR-M-0043192, SEM) **d***Milesinaexigua* on *Polystichumbraunii*, smooth surface (M, M-020547, SEM) **e***Milesinaexigua* on *Polystichumbraunii*, smooth surface, plasma-free spore, germ pores bipolar (M, M-0205472, LM, phase contrast) **f***Milesinafeurichii* on *Aspleniumseptentrionale* with smooth areas on surface (KR-M-0043159, SEM) **g***Milesinafeurichii* on *Aspleniumseptentrionale*, cracked plasma-free spore, germ pores scattered (KR-M-0043159, LM, phase contrast) **h***Milesinakriegeriana* on *Dryopteriscarthusiana* (KR-M-0048085, SEM) **i***Milesinamagnusiana* on *Aspleniumadiantum-nigrum* with smooth areas on surface (M, M-0205474, SEM) **j***Milesinamagnusiana* on *Aspleniumadiantum-nigrum*, spore plasma-free, germ pores scattered (M, M-0205474, LM, phase contrast) **k***Milesinamurariae* on *Aspleniumruta-muraria* with smooth areas on surface (KR-M-0035461, SEM) **l***Milesinamurariae* on *Aspleniumruta-muraria*, cracked spore with released plasma, germ pores scattered (KR-M-0043154, LM, phase contrast) **m***Milesinapolypodii* on *Polypodiumvulgare* with smooth areas on surface (KR-M-0043173, SEM) **n***Milesinascolopendrii* on *Aspleniumscolopendrium* with smooth areas on surface (KR-M-0049049, SEM) **o***Milesinavogesiaca* on *Polystichumaculeatum*, surface with very flat warts at the tip of the spore (arrow) (KR-M-0043160, SEM) **p***Milesinavogesiaca* on *Polystichumaculeatum*, surface smooth (no warts visible at the tip), germ pores bipolar (KR-M-0043175, LM, phase contrast) **q***Milesinawhitei* on *Polystichum* sp. (KR-M-0039378, SEM) **r***Milesinawhitei* on *Polystichumsetiferum*, cracked spore with released plasma, germ pores scattered (KR-M-0049177, LM, phase contrast).

### Milesina carpatica

Taxon classificationFungiPuccinialesPucciniastraceae

Wróbl., Sprawozdanie Komisji Fizjograficznej 47(II): 166 (1913)

Figure 6c

Dryopteris filix-mas (L.) Schott. Austria Steiermark: Graz, basilica Maria Trost, Rettenbachklamm, 11 Jun 1991, J. Poelt, II (GZU, GZU 000335631, GZU 000335632,); Koralpe, Wildbachgraben, ravine forest, 25 Apr 1988, J. Poelt, (M, M-0205477, M-0205478); Koralpe, Wildbachgraben, WNW Wildbach, NW Deutschlandberg, ravine forest, 25 Apr 1988, J. Poelt, II (GZU, GZU 000335634, GZU 000335635); Sausal-Gebirge, rift between Mitteregg and Voregg respectively Annaberg, 12 Apr 1981, J. Poelt, II (GZU, GZU 000335633); Germany, Bayern: Oberbayern, Murnau, Staffelsee, mixed forest, 15 Sep 2017, M. Scholler, II (KR, KR-M-0048589); Niedersachsen: Osterode, Bad Lauterberg, Barbis, forest, scarp, 30 Mar 2014, H. Thiel, II, III (KR, KR-M-0043192); Ukraine, Kolomyja: Knyazhdvir (formerly Galicia, Kniazdwor-Bania, Kolomea), Aug 1913, A. Wróblewski, II (M, M-0290298, type).

#### Description.

Urediniospores hyaline, ellipsoidal, obovoidal to subglobose, 16.5–32.5 × 10.0–20.0 µm, mostly 20.0–30.0 × 12.5–19.0 µm; wall 0.5–1.8 µm, mostly 0.5–1.0 µm thick; soft (in microscopic mounts they often crack without pressure), very densely echinulate without spine-free areas, spines 1.0–2.0 µm, mostly 1.0–1.8 µm long, irregularly distributed, spines typically straight and perpendicular to the wall, distance between spine bases 0.5–4.0 µm, mostly 0.5–3.0 µm, spine base 0.4–0.7 µm, mostly 0.5–0.6 µm; germ pores scattered, 4–10, mostly 5–7, 1.3–2.5 µm, mostly 1.3–2.5 µm diam., Ø 2.2 µm diam.

#### Comment.

Germ pores are more difficult to visualise and need more time to evaluate.

### Milesina exigua

Taxon classificationFungiPuccinialesPucciniastraceae

Faull, Contributions from the Arnold Arboretum of Harvard University 12: 218‒219 (1931)

Figure 6d, e

Polystichum aculeatum (L.) Roth (P.lobatum L., Aspidiumlobatum Sw.). Ukraine, Kolomyja: Knyazhdvir, Aug 1913, A. Wróblewski (as M.vogesiaca: Sydow, Uredineen 2742; GLM, GLM-53030; W, 1916-4289); Knyazhdvir, Sep 1913, A. Wróblewski (as M.vogesiaca: W, 1975-18645).
Polystichum braunii (Spenn.) Fée. Austria, Steiermark: Buchgraben NE Oberschöckl, canyon slope, 26 Apr 1983, J. Poelt, (GZU, GZU 000313869; M, M-0205472); Deutschlandsberg, Freiland, Wildbachklamm, south of the stream, scarp, 31 Jul 2018, M. Scholler & C. Scheuer (KR, KR-M-0050247); Höllgraben, ravine forest, 29 Sep 1988, J. Poelt & P. Zwetko (M, M-0205473). Koralpe, Mausegger-Graben next Sauerbrunn, NW Stainz, district Deutschlandsberg, ravine forest, silicate rock, 17 Apr 1995, J. Poelt (GZU, GZU 000313870); NW Stainz, Höllgraben WNW Marhof, ravine forest, 29 Sep 1988, J. Poelt & P. Zwetko (GZU, GZU 000313867).

#### Description.

Urediniospores hyaline, ellipsoidal to obovoidal, clavate, 22.5–32.5 × 12.5–17.5 µm, mostly 22.5–30.0 × 12.5–17.5 µm; wall 0.5–0.8 µm; spores smooth, germ pores low in number, probably around 4–6, 2.0–3.8 µm, mostly 2.0–3.0 µm diam., Ø 2.7 µm diam.; germ pores mostly apically, or both, basally and apically (bizonate).

#### Comment.

Klenke and Scholler (2015: 649) list this species (as *M.neoexigua* Berndt) on *Polystichumbraunii* for Germany (Baden-Württemberg), based on a specimen from the Black Forest, SW Germany (KR-M-0019138). *P.braunii* is a rare member of the southern Black Forest flora. We revised host and fungus and found that it is *M.vogesiaca* on *P.aculeatum*. Thus, the presence of *M.exigua* in Germany has not been confirmed. Germ pores are more difficult to visualise and need more time to evaluate.

### Milesina feurichii

Taxon classificationFungiPuccinialesPucciniastraceae

(Magnus) Grove, Journal of Botany 59: 311 (1921)

Figure 6f, g

Asplenium septentrionale (L.) Hoffm. Germany, Hessen: Werra-Meißner, Eschwege, Albungen, rock, 13 Apr 2013, H. Thiel, II (KR, KR-M-0043159); Sachsen: Vogtland, NNW Jocketa, NSG Steinicht, 9 May 1999, H. Jage & F. Klenke, II (GLM, GLM-F103536); Switzerland, Ticino: Locarno, Sciarana, Cugnasco, vineyard, dry stone wall, 9 Dec 2017, L. Beenken (KR, KR-M-0044955).

#### Description.

Urediniospores hyaline, ellipsoidal, obovoidal to subglobose, 27.5–42.5 × 17.5–25.0 µm, mostly 30.0–37.5 × 20.0–22.5 µm; wall 0.5–1.8 µm, mostly 0.5–1.0 µm thick; spores densely echinulate with 1–2, mostly 1 round to ovoidal smooth area, typically located centrally, smooth area 7.5–17.5 × 6.5–10.0 µm, mostly 10.0–15.0 × 7.5–10.0 µm, spines 1.5–2.5 µm, mostly 1.8-2.2 µm long, irregularly distributed, spines typically straight and perpendicular to the wall, distance between spine bases 1.0–9.0 µm, mostly 1.0–5.0 µm, spine base mostly around 1 µm; germ pores scattered, 5–11, mostly 6–7, 1.3–3.0 µm, mostly 2.0–2.5 µm diam., Ø 2.4 µm diam.

### Milesina kriegeriana

Taxon classificationFungiPuccinialesPucciniastraceae

(Magnus) Magnus, Bulletin de l’Institute et de jardin botanique de L’Université de Beograd 27: 325 (1909)

Figure 6h

Dryopteris borreri (Newman) Oberholzer & Tavel. Germany, Thüringen: Eichsfeld, Hundeshagen, forest stream canyon, 20 May 2014, H. Thiel (KR, KR-M-0043164).
D. carthusiana (Vill.) H.P. Fuchs. (= Aspidiumspinulosum Sw.). Germany, Sachsen: Bad Schandau, Schrammsteine, Sep 1893, Wegener, II (B, B 700016500); Sächsische Schweiz, 2 Nov 1901, W. Krieger, II (B, B 700016501); Uttewalder Grund, Oct and Nov 1901, W. Krieger (Krieger, Fungi Sax. Exs. 1711; HBG, 1/2338, 2/2338, 3/2338, type); Uttewalder Grund, Nov 1901, P. Magnus (B, B 700016499); Uttewalder Grund, Nov 1901, W. Krieger, II (B, B 700016498); Polenzthal (Polenztal), Königstein (Elbe), Sep 1901, W. Krieger, II (B, B 700016497); Sachsen-Anhalt: Burgenland, Wischroda, Braunsroda, 8 Okt 2013, H. Jage, II (KR, KR-M-0048086); Stendal, Gollensdorf, pine forest, 19 Oct 2014, H. Zimmermann (KR, KR-M-0048085); Wittenberg, Kemberg, Graditz, pine forest, 22 Feb 2014, H. Jage, II (KR, KR-M-0048087); Lüchow-Dannenberg, Dannenberg (Elbe), pine forest, 18 Mar 2014, H. Thiel (KR, KR-M-0043170).
D. dilatata (Hoffm.) A. Gray. Germany, Baden-Württemberg: Schwarzwald, Seebach, wayside, 4 Apr 2017, M. Scholler & M. Wieners (KR, KR-M-0048477); Ortenau, Seebach, NSG “Wilder See-Hornisgrinde”, spruce-fir forest, wayside, 4 Apr 2017, M. Scholler & M. Wieners (KR, KR-M-0048480); Hessen, Werra-Meißner, Lichtenau Hoher Meißner, broadleaved forest, 13 Mar 2014, H. Thiel (KR, KR-M-0043162); Mecklenburg-Vorpommern, Rügen, Putbus, Vilm, forest, 20 Aug 2014, H. Thiel (KR, KR-M-0039321); Rügen, Putbus, Vilm, 20 Aug 2014, S. Hoeflich, H. Jage, II (KR, KR-M-0048357); Niedersachsen, Göttingen, Landolfshausen, Potzwenden, douglas fir-spruce forest, 18 Jan 2014, H. Thiel (KR, KR-M-0043182); Lüchow-Dannenberg, Küsten, Sallahn, pine forest, 10 Nov 2013, H. Thiel (KR, KR-M-0043151); Northeim, Hardegsen, Ertinghausen Meinheitsberg, spruce forest, 21 Jun 2014, H. Thiel (KR, KR-M-0043165); Harz, Osterode, Herzberg, Lonau, spruce forest, 9 Aug 2012, H. Thiel (KR, KR-M-0043184); Sachsen-Anhalt, Wittenberg, Schköna, pine forest, 27 Feb 2014, H. Jage, II (KR, KR-M-0048088).
D. filix-mas (L.) Schott. Germany, Niedersachsen: Northeim, beech forest, 16 Jan 2014, H. Thiel (KR, KR-M-0043178); Sachsen (?): Bad Schandau?, 1895, G. Wagner, II (HBG, 4/2338).

#### Description.

Urediniospores hyaline, ellipsoidal, obovoidal to oval, clavate, 25.0–47.5 × 12.5–25.0 µm, mostly 27.5–37.5 × 15.0–20.0 µm; wall 0.5–1.2 µm, mostly 0.8–1.0 µm thick; spores echinulate without spine-free areas, spines 1.2–3.0 µm, mostly 1.8–2.2 µm long, irregularly distributed, sometimes in rows, spines typically straight and perpendicular to the wall, distance between spine bases 1.0–6.0 µm, mostly 1.0–4.0 µm, mostly around 1 µm; germ pores scattered, 6–14, mostly 10–11, 1.3–3.0 µm, mostly 2.0–2.5 µm, Ø 2.3 µm diam.

#### Comment.

See annotation under *M.blechni*.

### Milesina magnusiana

Taxon classificationFungiPuccinialesPucciniastraceae

Jaap, Verhandlungen des Botanischen Vereins für die Provinz Brandenburg 57: 16 (1915)

Figure 6i, j

Asplenium adiantum-nigrum L. France, La Corse: Ajaccio, 5 Mar 1933, O. Jaap, II (M, M-0290299, type); Ireland: Kerry, Dingle peninsula, drywall, 30 Aug 1964, Leuze & Doppelbaur (M, M-0205474).

#### Description.

Urediniospores hyaline, ellipsoidal to obovoidal, 21.3–38.8 × 15.0–22.5 µm, mostly 30.0–35.0 × 17.5–20.0 µm; wall 1.0–2.0 µm, mostly 1.0–1.5 µm thick; spores echinulate with 1–2 ovoidal smooth areas, typically located centrally, smooth area 11.5–17.5 × 6,3–10.0 µm, mostly 15.0–17.5 × 7.5–10.0 µm, spines 1.2–2.8 µm, mostly 2.0–2.2 µm long, irregularly distributed, spines often erect, distance between spines 0.5–9.0 µm, mostly 3.0–5.5 µm; germ pores scattered, 4–9, mostly 5–6, 2.0–4.5 µm, mostly 2.5–3.0 µm diam., Ø 2.9 µm diam.

### Milesina murariae

Taxon classificationFungiPuccinialesPucciniastraceae

(Magnus) Grove, Journal of Botany, London 59: 311 (1921)

Figure 6k, l

Asplenium ruta-muraria L. Austria, Tirol: Landeck, 22 Feb 1900, O. Jaap, II (HBG, 7/2338); Bad Ratzen, 23 Aug 1908, P. Magnus, II (HBG, 13/2338); Innsbruck, near Klausen, 21 Aug 1902, P. Magnus, II (HBG, 14/2338); Vorarlberg: Bludenz, 21 Aug 1909, P. Magnus, II (HBG, 8/2338); Salzburg: Zell am See, 1 Sep 1890, P. Magnus, II (HBG, 6/2338); France, Alsace: Forbach, Melponte, 12 Jul 1912, A. Ludwig, II (GLM, GLM-53031); Germany, Baden-Württemberg: Freiburg/Breisgau, St. Peter, wall of monastery, 22 Aug 1999, H. Jage (KR, KR-M-0025191); Bodensee, Konstanz, Stockach, “Am Stadtwall”, wall, 29 Aug 2000, H. Jage (KR, KR-M-0025185); Bodensee, Konstanz, Stockach, Oberstadt “Am Stadtwall”, wall, 27 Aug 2001, H. Jage (KR, KR-M-0025768); Tübingen, downtown, wall, 3 Jul 2008, H. Jage (KR, KR-M-0036224); Tübingen, Bebenhausen monastery, 2 Jul 2008, H. Jage (KR, KR-M-0036225); Sigmaringen, Beuron, monastery wall, 29 Jul 2000, H. Jage (KR, KR-M-0025184); Niedersachsen: Hildesheim, town wall, W women’s prison, 1 Mar 2013, H. Thiel (KR, KR-M-0043149); Osterode, Bad Grund, chalk rocks, 16 Jan 2014, H. Thiel (KR, KR-M-0043154); Sachsen: Erzgebirge, Zschopautal, Apr 1902, G. Wagner, (HBG, 5/2338); Sachsen-Anhalt: Spielberg, Burgenlandkreis, Benndorf, wall of churchyard, 2 Jun 2014, H. Jage (KR, KR-M-0048134); Wittenberg, Kemberg, city wall, 13 Jul 2014, H. Jage (KR, KR-M-0048133); Thüringen: Saale-Holzland, Dornburg/Saale, cemetery wall, 21 Oct 2012, H. Jage & G. Vogel (KR, KR-M-0035461); Weimarer Land, Apolda, wall, 9 Oct 2013, H. Jage & G. Vogel, (KR, KR-M-0048132); Sachsen: Wehlen, 12 May 1911, W. Krieger (HBG, 21/2338); Italy, Alto Adige: Voldertal, next to Kreuz Häusle, 8 Sep 1910, P. Magnus, II (HBG, 9/2338); Bolcano, vineyard wall, 4 Sep 1890, P. Magnus, II (HBG, 10/2338); Bolcano, Waidbruck between Tergolerbrücke and Kollmann, 15 Jul 1907, A. Heimerl (HBG, 12/2338); Bolcano, Seis am Schlern, 20 Aug 1908, P. Magnus, II (HBG, 16/2338); Seis am Schlern, 26 Aug 1908, P. Magnus, II (HBG, 18/2338); garden wall in Vahrn, 6 Jul 1905, A. Heimerl, II (HBG, 15/2338 Merano, 14 Sep 1890, P. Magnus, II (HBG, 11/2338); Intra, Lago Maggiore, 18 Sep 1892, P. Magnus, II (HBG, 19/2338); Trentino: St. Cristoforo, Val Lugano, 27 Aug 1910, P. Magnus, II (HBG, 17/2338); Switzerland, Bern: Twann, at Bielersee, 12 Mai 1909, E. Fischer, II (HBG, 20/2338).

#### Description.

Urediniospores hyaline, ellipsoidal, obovoidal to subglobose, 25.0–42.5 × 15.0–22.5 µm, mostly 27.5–35.0 × 17.5–22.5 µm; wall 1.2–2.2 µm, mostly around 2.0 µm thick; spores echinulate with 1–2, mostly 2 ovoidal smooth areas, typically located centrally, smooth area 11.5–20.0 × 7.5–12.5 µm, mostly 12.5–15.0 × 7.5–10.0 µm, spines 1.5–2.5 µm, mostly 1.8–2.2 µm long, erect, spines curved toward base, denser toward both spore poles, distance between spine bases 0.5–7.0 µm, mostly 2.0–3.5 µm, spine base 0.7–1.4 µm, mostly around 1 µm; germ pores scattered, 3–9, mostly 5–6, 2.0–3.8 µm, mostly 2.0–2.5 µm diam., Ø 2.4 µm diam.

### Milesina polypodii

Taxon classificationFungiPuccinialesPucciniastraceae

(F.B. White) Aime & Rossman, in Aime, Castlebury, Abbasi, Begerow, Berndt, Kirschner, Marvanova, Ono, Padamsee, Scholler, Thines & Rossman, IMA Fungus 9(1): 83 (2018)

Figure 6m

Polypodium interjectum Shivas. France, Alsace: Wasselnheim, Wangenberg, 23 Oct 1914, A. Ludwig, II (W, 1916-3467); way Fischboedle to Hohneck, 3 Jul 1910, H. Sydow, II (S, F310825); Potigny (Calvados), Bréche-au-Diable, 14 Apr 1911, R. Maire, II (W, 1912-3055; B, B 700016502); Germany, Nordrhein-Westfalen: Märkischer Kreis, Balve, Volkringhausen, moist forest, 16 Aug 2012, H. Thiel, II (KR, KR-M-0043189).
P. × mantoniae Rothm. & U. Schneid. (P.vulgare L. × P.interjectum Shivas). Germany, Niedersachsen: Northeim, SO Vorwerk Levershausen, Langfast Kopf, broadleaved forest, sandstone, 16 Jan 2014, H. Thiel, II (KR, KR-M-0043177).
P. vulgare L. Germany, Baden-Württemberg: Schwarzwald, Ortenau, Lautenbach, Lautenfelsen, 5 Jun 2017, M. Scholler & A. Rubner, II (KR, KR-M-0048694); Schwarzwald, Ortenau, Ottenhöfen, slope near creek, next to Abiesalba, 13 Nov 2017, M. Scholler & R. Buchheit (KR, KR-M-0049181); Schwarzwald, Ortenau, Ottenhöfen, scarp near stream, coniferous forest next to Abiesalba, 13 Nov 2017, M. Scholler & R. Buchheit (KR, KR-M-0049179); Hessen: Hessisch Lichtenau, Hoher Meißner, border area of an open “Basaltblockhalde”, 13 Mar 2014, H. Thiel (KR, KR-M-0043161); Niedersachsen: Ammerland, Bad Zwischenahn, Ofen, mixed forest, 11 Apr 2017, R. Jarling, II (KR, KR-M-0048818); Lüchow-Dannenberg, Höbeck, beech-oak forest, 22 Dec 2013, H. Thiel, II (KR, KR-M-0043146); Rheinland-Pfalz: Bad Kreuznach, Hochstetten-Dhaun, Simmerbachtal near Dhaun castle of Dhaun, 30 Oct 2013, H. Thiel (KR, KR-M-0043152); Schleswig-Holstein: Nordfriesland, Nebel, pine forest, 15 Oct 2014, H. Thiel (KR, KR-M-0043173); Sachsen: Schmilka, Großer Winterberg, 26 Aug 1903, H. & P. Sydow (Sydow & Sydow, Mycoth. germ. 62; W, 1903-13407; S, F29305, type); Schmilka, Großer Winterberg, Aug 1903, H. & P. Sydow (S, F29306, F29307, type); Sachsen-Anhalt, Quedlinburg, Thale, unteres Bodetal, beneath Hexenplatz, 17 Jul 2014, H. Thiel (KR, KR-M-0043157); Thüringen, Eisenach, SSW Wartburg, NNW Eisenacher Burg, 25 May 2012, H. Thiel (KR, KR-M-0043190); Great Britain, Wales: Harlech, 4 Jan 1927, P.G.M. Rhodes (W, 1975-15143, 1973-22169, 1973-22320); Romania, Vîlcea: Muntele Cozia, Omu, 1 Jul 1976, G. Negrean, II (W, 1980-00055); Switzerland, Neuenburg: Gorgier, Creux du Van, 19 Oct 1913, E. Mayor, II (W, 1914-9288); Neuchâtel, Bois, Tête-Plumée, 5 Nov 1909, E. Mayor, (Vestergren, Micromyc. rar. sel. praec. Scand. 1702, W, 1914-9287, 1973-17308,); Neuchâtel, Tête-Plumée, 20 Oct 1913, E. Mayor, II (W, 1915-5830).

#### Description.

Urediniospores hyaline, ellipsoidal, obovoidal to subglobose, 26.5–42.5 × 15.0–25.0 µm, mostly 30.0–40.0 × 17.5–22.5 µm; wall 0.5–2.5 µm, mostly 0.5–1.0 µm thick; spores echinulate with 1–2, mostly 1 ovoidal smooth area, typically located centrally, smooth area 15.0–22.5 × 6.3–11.3 µm, mostly 15.0–17.5 × 7.5–10.0 µm, spines 1.8–2.8 µm, mostly 1.8–2.2 µm long, irregularly distributed, erect, spines denser toward spore base, distances 0.5–7.0 µm, mostly 1.0–4.0 µm, spine base 0.7–1.6 µm, mostly 0.9–1.2 µm diam.; germ pores scattered, 4–10, mostly 5–6, 1.3–3.8 µm, mostly 2.0–2.5 µm diam., Ø 2.3 µm diam.

### Milesina scolopendrii

Taxon classificationFungiPuccinialesPucciniastraceae

(Fuckel) Jaap, Fungi selecti exsiccati no. 571 (1912)

Figure 6n

Asplenium scolopendrium L. (Phyllitisscolopendrium (L.) Newman). Germany, Baden-Württemberg: Bodensee, Konstanz, N Langenrain, Überlinger-See, NW part of Blisenhalde, 9 May 1999, H. Jage (KR, KR-M-0025400); Reutlingen, Bad Urach, 12 Sep 2017, M. Scholler & R. Buchheit (KR, KR-M-0049049, KR-M-0049050); Reutlingen, Bad Urach, Gütersteiner Wasserfall, 18 Sep 2017, R. Buchheit (KR, KR-M-0049066); Reutlingen, Bad Urach, Uracher Wasserfälle, 12 Sep 2017, M. Scholler & R. Buchheit (KR, KR-M-0049037, KR-M-0049051); Nordrhein-Westfalen, Märkischer Kreis, Balve, Volkringhausen, moist forest, limestone rocks, 16 Aug 2012, H. Thiel (KR, KR-M-0043186); Rheinland-Pfalz, Bad Kreuznach, Hochstetten-Dhaun, forested slope below Dhaun castle, 30 Oct 2013, H. Thiel, II (KR, KR-M-0043153); Switzerland, Graubünden: 1871-1873, L. Fuckel (G, G 00550757, type).

#### Description.

Urediniospores hyaline, ellipsoidal to obovoidal, clavate, 27.5–49.0 × 17.5–25.0 µm, mostly 27.5–42.5 × 17.5–22.5 µm; wall 0.5–1.8 µm, mostly 0.5–1.2 µm thick; spores echinulate with 1 mostly ovoidal smooth area, located centrally to apically, smooth area 12.5–20.0 × 7.5–11.3 µm, mostly 15.0–17.5 × 7.5/10.0 µm, spines 1.5–2.8 long, irregularly distributed, erect, distances between spine bases 1.0–9.0 µm, mostly 2.0–5.0 µm, sometimes denser toward spore base, spine base 0.8–1.6 µm, mostly 0.9–1.2 µm diam.; germ pores scattered, 4–9, mostly 6–7, 1.25–3.0 µm, mostly 2.0–3.0 µm diam., Ø 2.4 µm diam.

### Milesina vogesiaca

Taxon classificationFungiPuccinialesPucciniastraceae

Syd. & P. Syd., Annales Mycologici 8(5): 491 (1910)

Figure 6o, p

Polystichum aculeatum (L.) Roth. (P.lobatum L, Aspidiumlobatum Sw.). Austria, Kärnten: Hermagor, between upper and lower Valentin Alpe next to Mauthen, 26 Aug 1940, H. Poeverlein (W, 1973-28256); Tirol: Alps, western Elbigenalp, Bernhardstal toward Bernhardseck, ravine forest, 28 Aug 1992, H. Jage, II (GLM, GLM-50893); France, Alsace: Lützelhausen, 5 Dec 1914, A. Ludwig, II (GLM, GLM-53028; W, 1916-4290); between Fischboedle and Kerbholz, Hohneck, 12 Jul 1910, H. Sydow (Sydow & Sydow, Mycoth. germ. 878; W, 1973-30304, 1910-006973; S, F29337, F29338, 29339, type); Vosges, between Fischboedle and Kerbholz, Hohneck, 16 Jul 1910, H. Sydow (Sydow, Uredineen 2345; B, B 700016496; W, 1973-07263, 1911-3905, type); between Fischboedle and Kerbholz, Hohneck, 16 Jul 1910, H. Sydow (S, F310782; B, B 700016495, type); Germany, Baden-Württemberg: Todtmoos, Au, Wehratal, Hagenmattgraben, 23 Aug 2001, H. Jage (sub M.neoexigua), corr. M. Scholler (KR, KR-M-0019138); Bayern: Oberallgäu, Oberjoch, Iseler, spruce-fir forest, 24 Jun 2008, M. Scholler (KR, KR-M-0003907); Hessen: Werra-Meißner, Eschwege, Albungen, Trenkgraben, broadleaved forest, 13 Apr 2013, H. Thiel (KR, KR-M-0043160); Niedersachsen, Osterode, Herzberg, Scharzfeld, Rottsteine, beech forest, limestone rocks, 29 Mar 2014, H. Thiel (KR, KR-M-0043175); Nordrhein-Westfalen: Märkischer Kreis, Balve, Volkringhausen, moist forest, 16 Aug 2012, H. Thiel, II (KR, KR-M-0043187).
P. lonchitis (L.) Roth. Austria, Tirol: Alps, Lechtal, south east Holzgau, Sulzltal, 1 km south of Ronig-Alm, “Hochstaudenbergflur” 17 Aug 1991, H. Jage, II (GLM, GLM-50866).

#### Description.

Urediniospores hyaline, ellipsoidal to obovoidal, clavate, 27.5–45.0 × 15.0–25.0 µm, mostly 30.0–40.0 × 17.5–20.0 µm; wall 0.5–1.0 µm, mostly 0.5–0.8 µm thick; spores with flat verrucae verrucae 0.3–0.6 µm, mostly 0.4–0.5 µm in diam., mainly at the upper part of the spore (visible with SEM only); germ pores often bizonate, sometimes scattered, 3–8 mostly 5–6, 2.0–4.5 µm, mostly 2.5–3.0 µm diam., Ø2.8 µm diam.

#### Comment.

See commentary under *M.exigua*.

### Milesina whitei

Taxon classificationFungiPuccinialesPucciniastraceae

(Faull) Hirats., Memoirs of the Tottori Agricultural College 4: 123 (1936)

Figure 6q, r

Polystichum aculeatum (L.) Roth (syn. P.lobatum L, Aspidiumlobatum Sw.). Croatia, Dalmatia: Castelnuovo, 25 Apr 1914, O. Jaap (FH, FH 01146298, type).
Polystichum setiferum (Forsk.) Moore ex Woynar., Austria, Steiermark: Deutschlandberg, Klause, Laßnitz, northern bank of the river, rock, 31 Jul 2018, M. Scholler & C. Scheuer (KR, KR-M-0050248); Switzerland, Ticino: Locarno, Cugnasco, Valle di Cugnasco, ravine, 9 Dec 2017, L. Beenken (KR, KR-M-0044953); Vaud, Montreux, Gorges du Chadron, 30 Apr 2011, T. Brodtbeck, II (KR, KR-M-0049177).
Polystichum sp. Austria, Steiermark: Possruck, ravine, 19 Nov 1972, J. Poelt (KR, KR-M-0039378).

#### Description.

Urediniospores hyaline, ellipsoidal, obovoidal to oval, 27.5–40.0 × 16.5–25.0 µm, mostly 27.5–37.5 × 17.5–22.5 µm; wall 0.5–1.0 µm, mostly 0.8–1.0 µm thick; echinulate without spine-free areas, spines 1.8–2.8 µm, mostly around 1.8–2.5 µm long, irregularly distributed, straight and perpendicular to the wall, distance between spine bases 1.0–8.0 µm, mostly 1.5–5.0 µm, spine base 0.5–1.2 µm, mostly 0.8–1.1 µm diam.; germ pores scattered, 8–15 (17), mostly 9–13, 1.3–3.0 µm, mostly 2.0–2.5 µm diam., Ø2.3 µm diam.

#### Comment.

The North American *Milesinapolystichi* (Wineland) Grove (= *Milesiapolystichi* Wineland) on *Polystichummunitum* (Kaufl.) Presl. is considered conspecific with *M.whitei* by several authors (e.g. Gäumann 1959). We were able to study isotype material (USA, Oregon, Granite Pass, 5 Sep 1916, leg. R.J. Weir, PUR 004047) and found urediniospores with mostly 5 to 6 germ pores, i.e. many fewer than in *M.whitei*. Due to this striking difference, they are possibly different species.

### Milesina woodwardiana

Taxon classificationFungiPuccinialesPucciniastraceae

Buchheit & M. Scholler sp. nov.

829596

Figure 7a–f

#### Holotype.

*Woodwardiaradicans* (L.) Sm., Spain, Islas Canarias, La Palma, Cubo de la Galga, ca. 2.5 km SW parking place at coastal highway W San Bartolomé, wayside in Laurosilva, 11 Aug 2017, V. Kummer (KR-M-0049033).

Further specimens examined (paratypes)Spain, Islas Canarias: La Palma, Cubo de la Galga, ca. 1.2 km SW of parking lot at coastal highway W San Bartolomé, wayside in Laurosilva, 16 Aug 2015, V. Kummer, II (KR, KR-M-0048787); La Palma, Cubo de la Galga, ca. 1.2 km SW parking lot at coastal highway W San Bartolomé, near waypoint 6, wayside in Laurosilva, 11 Aug 2017, V. Kummer, II (KR, KR-M-0049036); La Palma, Cubo de la Galga, ca. 1.25 km SW parking lot at coastal highway W San Bartolomé, between waypoint 6 and 7, wayside in Laurosilva, 11 Aug 2017, V. Kummer, II (KR, KR-M-0049034).

#### Description.

Spermogonia (0), aecia (I), telia (III) and basidia (IV) unknown. Uredinia hypophyllous, subepidermal, statistically distributed; sori round, wart-like elevations, 0.1–0.3 mm in diam., covered by brownish or yellow-brownish epidermis, on dark necrotic plant tissue margined by nerves, never on nerves directly, sori opening pore-like; peridium hemispheric, peridial cells colourless, about 7.5–25.0 × 7.5–10 µm, upper peridial cells more or less isodiametrical and lateral peridial cells elongated; urediniospores hyaline, ellipsoidal to obovoidal, sometimes subglobose to irregular, 25.5–46.5 × 15.0–25.0 µm, mostly 30.0–37.5 × 17.5–22.5 µm; cell wall thin, 0.5–1.2 µm, mostly 0.5–1.0 µm thick, densely echinulate without spine-free areas, densest at spore base, spines 2.0–3.2 µm long, mostly 3.0 µm long, slightly irregularly distributed, spines orientated in different directions, dense basal spines typically directed toward spore pedicel, distance between spines bases 0.5–5.0 µm, mostly 2.0–4.0 µm, spine base 0.6–1.3 µm, mostly around 1 µm; spore pedicel often laterally or semilaterally inserted, short and wide, 5.5–14 × 12.5–15.5 µm; germ pores scattered, 8–19 (21), mostly 10–14, 1.3–3.0 µm, mostly 2.0–3.0 µm diam., Ø 2.4 µm diam.; germ tubes septate, may develop simultaneously in one spore.

**Figure 7.**
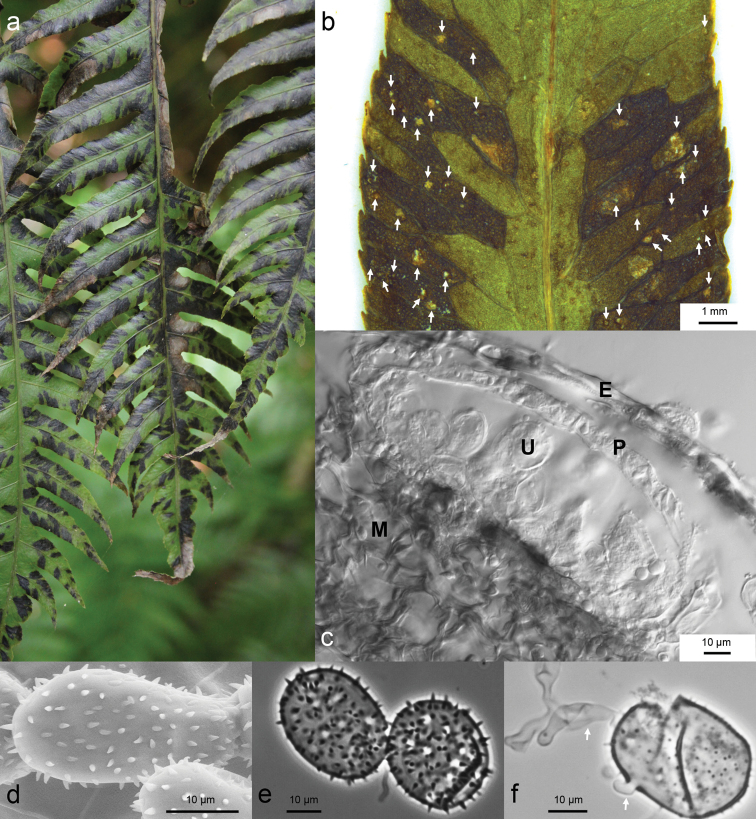
Spore morphology and symptoms on fern fronds of *Milesinawoodwardiana* sp. nov. **a** Fronds of the host *Woodwardiaradicans* at the collection site in La Palma. Dark spots indicate areas where sori are formed on the underside (La Palma, Cubo de la Galga, ca. 1.2 km SW of parking lot W San Bartolomé, 11 Aug 2017) **b** Host leaf with uredinia. Sori (arrows) are restricted to areas between leaf veins (KR-M-0048787, dissecting microscope) **c** Transverse section of uredinium E=epidermis, P=peridial cells, U=urediniospore, M=mesophyll of host plant (KR-M-0048787, LM, interference contrast) **d** Urediniospores with long echinulae (KR-M-0049036, paratype, SEM) **e** Urediniospores, cracked, without plasma, germ pores scattered (KR-M-0049033, paratype; LM, phase contrast) **f** Germinating urediniospores, arrows point to germ tubes (KR-M-0049033, paratype, LM, phase contrast).

#### Distribution.

The species is only known north-eastern La Palma, Islas Canarias, Spain.

#### Etymology.

Referring to the English botanist Thomas Jenkinson Woodward (1745 – 1820) and the host plant *Woodwardiaradicans* named after him.

#### Comment.

This species differs from *M.blechni* by the telial host plant genus (*Woodwardia*), by a higher number of germ pores/spore, longer spines and irregular spine orientation. *Milesinawoodwardiana* is the first *Milesina* species known on *Woodwardia* (Berndt 2008; Faull 1932). The absence of potential aecial hosts (*Abies* spp.) in La Palma and all other Canary Islands (Hohenester and Welss 1993, Ginovés et al. 2009) and the non-formation of telia indicate that the species is not host-alternating in La Palma. The *Woodwardiaradicans* area (Hohenester and Welss 1993), however, overlaps with those of *Abies×borisii-regis*, *A.cephalonica* and *A.pinsapo* in south-western Europe (Liu 1971). If the rust is present in this area, it may be possible to observe the spore stages 0 and I on *Abies* spp. *Woodwardiaradicans* is the only species of *Woodwardia* in Europe. There are numerous other species in SE Asia and N America (Li et al. 2016). These areas may also coincide with the distribution area of *M.woodwardiana*.

### Milesina spp.

Taxon classificationFungiPuccinialesPucciniastraceae

Abies alba Mill. Austria, Steiermark: Trog, Mausegg, Höllental, Klamm, N river bank, 31 Jul 2018, M. Scholler & C. Scheuer, 0, I (KR, KR-M-0050303); Germany, Baden-Württemberg: Esslingen, Kirchheim unter Teck, NW Owen, 18 Sep 2017, R. Buchheit, I (KR, KR-M-0049065); Freudenstadt, Baiersbronn, NSG “Wilder See-Hornisgrinde”, Karwand, mixed spruce-fir forest, 12 Sep 2015, M. Scholler, I (KR, KR-M-0043687); Ortenaukreis, Friesenheim, E Oberweier near Schnaiggraben, mixed forest, 17 Aug 2005, M. Scholler (KR, KR-M-0018587); Ortenaukreis, SSW Oppenau, “Ibacher Holzplatz“ mixed spruce-fir forest, 18 Aug 2005, M. Scholler (KR, KR-M-0018624); Ottenhöfen, “Edelfrauengrab Wasserfälle”, creek bank, 26 Jun 2017, M. Scholler (KR, KR-M-0048773); Rastatt, Gernsbach, Lauterbach, NSG “Lautenfelsen“, eastern crosscut, beyond “Aussichtfelsen”, 11 Sep 2014, M. Scholler (KR, KR-M-0042052); Rastatt, Loffenau, “Großes Loch“ near Loffenau, 15 Sep 2017, R. Buchheit (KR, KR-M-0049038); Reutlingen, W Bad Urach, “Tannenverjüngung“, 18 Sep 2017, R. Buchheit, I (KR, KR-M-0049068); Bayern: Garmisch-Partenkirchen, Grainau, S Eibsee, spruce-fir forest, 8 Aug 2017, M. Scholler (KR, KR-M-0049063); Miesbach, Schliersee, S Spitzingsee, E Roßkopf, spruce-fir forest close to Struthiopterisspicant, 8 Aug 2017, M. Scholler (KR, KR-M-0049062).

#### Comment.

In this study, *Milesina* spp. on *Abiesalba* were only sequenced but not morphologically analysed.

##### Subgeneric classification

Four morphological groups can be distinguished within *Milesina* with respect to germ pore number, germ pore size, germ pore position and distribution of spines on the spore surface (Figures 6a–r, 7d–f, Table. 4). The morphological differentiation corresponds with the differentiation in four clades found by molecular data (Figure. 1, right panel).

### Milesina sect. Milesina

Taxon classificationFungiPuccinialesPucciniastraceae

#### Type species.

*M.kriegeriana* (Magnus) Magnus 1909.

This type section is characterised by urediniospores having numerous scattered germ pores and an echinulate wall without smooth areas. *Milesinablechni*, *M.whitei* and *M.woodwardiana* are additional members of this section.

### Milesina sect. Vogesiacae

Taxon classificationFungiPuccinialesPucciniastraceae

M. Scholler & Bubner sect. nov.

829594

#### Type species.

*M.vogesiaca* Syd. & P. Syd. 1912.

This section is characterised by urediniospores having few bipolarly distributed germ pores and a smooth or almost smooth wall. *Milesinaexigua* is included in this section. Urediniospore features of European *Uredinopsis* spp. resemble those of *Vogesiacae* species. However, *Uredinopsis* spores have a terminal mucro.

### Milesina sect. Scolopendriorum

Taxon classificationFungiPuccinialesPucciniastraceae

M. Scholler & Bubner sect. nov.

829597

#### Type species.

*M.scolopendrii* (Fuckel) Jaap 1912.

This section is characterised by urediniospores having few scattered germ pores and an echinulate wall with smooth areas. *Milesinafeurichii, M.polypodii*, *M.magnusiana* and *M.murariae* are in this section. *M.magnusiana* agrees well with the other species with respect to morphology. Therefore, we placed it in section Scolopendriorum, although no ITS data are available.

### Milesina sect. Carpaticae

Taxon classificationFungiPuccinialesPucciniastraceae

M. Scholler & Bubner sect. nov.

829595

#### Type species.

*M.carpatica* Wróbl. 1913

This section is characterised by urediniospores having few scattered germ pores and an echinulate wall without smooth areas. It is similar to section Milesina in having an echinulate cell wall, but the number of germ pores is lower (only 5-7). The ITS sequences of the two sections are separated by a large genetic distance. So far, this section is represented only by the type species. Possibly, the North American *M.polystichi* belongs to this section as well (see commentary to *M.whitei*).

### Key to European *Milesina* species

The following key to European *Milesina* sections and species is based on urediniospore (abbreviated Us) features listed in Table 4. It requires light-microscopical equipment and methods described in the Methods section. The lengths of the urediniospores refer to the main values.

**Table:** 

1	Us with terminal mucro	*** Uredinopsis ***
–	Us without terminal mucro (*Milesina*)	**2**
2	Surface of Us smooth or almost smooth, germ pores often formed apically (sect. Vogesiacae)	**3**
–	Surface of Us echinulate, sometimes with particularly smooth areas, germ pores scattered	**4**
3	Us mostly 30.0–40.0 × 17.5–20.0 µm, germ pores up to 4.5 µm diam. (*Polystichumaculeatum*)	*** M. vogesiaca ***
–	Us smaller, mostly 22.5–30.0 × 12.5–17.5 µm, germ pores smaller, up to 3.8 µm diam. (germ pores are often not visible, check numerous Us) (*Polystichumbraunii, P.aculeatum*)	*** M. exigua ***
4	Surface of Us with smooth spine-free areas, germ pores ± 6 (Sect. Scolopendriorum)	**5**
–	Surface of Us without smooth spine-free areas, germ pores either ± 6 (*M.carpatica*, sect. Carpaticae) or ± 11 (species of sect. Milesina)	**9**
5	Us mostly 27.5–35.0 µm long, wall mostly 2.0 µm thick (*Aspleniumruta-muraria*)	*** M. murariae ***
–	Us mostly more than 30.0 µm long, wall mostly thinner (< 2 µm)	**6**
6	Us mostly 30.0–40.0 µm long (*Polypodium* spp.)	*** M. polypodii ***
–	Us shorter, mostly 30.0‒37.5 µm	**7**
7	Spine distance mostly 1.0‒4.0 µm (*Aspleniumseptentrionale*)	*** M. feurichii ***
–	Spine distance 2.0‒5.5 µm	**7**
8	Spine distance mostly 3.0‒5.5 µm, Us 30.0–35.0 × 17.5‒20.0 µm, germ pore 2.9 µm diam. (*Aspleniumadiantum-nigrum*)	*** M. magnusiana ***
–	Spine distance 2.0‒5.0 µm, Us 27.5–42.5 × 17.5–22.5 µm, germ pore < 2.5 diam. (*Aspleniumscolopendrium*)	*** M. scolopendrii ***
9	Us mostly 20.0‒30.0 × 12.5‒19.0 µm, wall 0.5‒1.0 µm thick, spines mostly 1.0‒1.8 µm long, germ pores usually 6, mostly 1.3‒2.0 µm diam., pores hardly visible (check numerous Us) (*Dryopterisfilix-mas*) (sect. Carpaticae)	*** M. carpatica ***
–	Us larger, mostly 27.0 ‒ 37.5 × 17.5 ‒ 22.5 µm, germ pores ± 11	**9**
10	Spines ± 3.0 µm long, orientated in different directions, Us mostly 30.0‒37.5 × 17.5‒22.5 µm (*Woodwardiaradicans*)	*** M. woodwardiana ***
–	Spines shorter, < 3.0 µm long, typically perpendicular to the wall	**11**
11	Us mostly ≥ 17.5 µm wide, 27.5‒40.0 × 16.5–25.0 µm, spines erect (*Polystichumaculeatum*, *P.setiferum*)	*** M. whitei ***
–	Us ± 17.5 µm wide, sometimes spines arranged in rows, typically erect (the following two species are morphologically barely distinguishable)	**12**
12	Us mostly 27.5–37.5 µm long (*Dryopterisborreri*, *D.carthusiana*, *D.dilatata*, *D.filix-mas*	*** M. kriegeriana ***
–	Very similar, Us somewhat longer on average, mostly 30.0–37.5 µm (*Struthiopterisspicant*)	*** M. blechni ***

## Discussion

### Species resolution by urediniospore features

In previous studies of the genus *Milesina* (e.g. Wróblewski 1913; Faull 1932; Kuprevič and Tranzschel 1957; Berndt 2008), size and shape of urediniospores, wall thickness, spine length and density were the main features used to characterise their morphology. In this study, additional morphological features and criteria are provided to distinguish species (Figure 5, Table 4).

The number, position and size of germ pores have not been documented even in more recent studies of *Milesina* (Berndt 2008). Additionally, in *Chrysomyxa*, another genus of Pucciniastraceae (Cao et al. 2017), no germ pores were shown. Germ pores in *Milesina* are documented in Cummins and Hiratsuka (2003). The authors report “bizonate, obscure” germ pores for species of the genus. We found this character only in the two species of the section Vogesiacae. A further observation of germ pores is reported for two North American species *Milesinapolypodophila* (Bell) Faull and *Milesinamarginalis* Faull & Watson (Moss 1926) where germ pores showed a scattered distribution. With our light microscopic method, detection of germ pores was easy and could be realised within short time. In two species, *M.carpatica* and *M.exigua*, germ pores were more difficult to visualise and need more time to evaluate. In general, however, this method is suitable to document an important morphological and taxonomically relevant feature. It may also help to characterise other genera in the Pucciniastraceae. Another feature, smooth areas on the surface of urediniospores has not been documented so far. It is a special character of species of section Scolopendriorum. All of these features in combination allow identification of *Milesina* species in Europe by urediniospore features alone, using a light microscope even without knowledge of the host plant species. Only one pair of species, the common *M.blechni* and *M.kriegeriana*, is difficult to distinguish morphologically. We only found differences in spore length measurements.

In general, identification using only the host is unreliable, since the range of telial hosts in *Milesina* has been only scarcely studied. This holds true even for common species like *M.kriegeriana*, a species which has obviously a much wider host range with species in different host families (Berndt 2008) than listed in European compilatory literature (e.g. Gäumann 1959; Majewski 1977; Klenke and Scholler 2015). *Polystichumaculeatum* is known for hosting *M.carpatica* and *M.vogesiaca* (e.g. Gäumann 1959; Majewski 1977; Klenke and Scholler 2015). In the present study, *M.exigua* was also found on *P.aculeatum*, demonstrating again that the host range may be wider for several *Milesina* species and that host identification is not sufficient to identify *Milesina* spp.

### Species resolution by barcoding

We were able to classify four sections by phylogenetic analysis of ITS sequences (Fig. 1) but we were only able to differentiate 4 of 11 species. This low differentiation is in contrast to another genus in the suborder Melampsorineae sensu Aime (2006). In the genus *Chrysomyxa*, almost all species could be resolved on the basis of ITS barcodes (Cao et al. 2017). Still, amongst the three tested barcodes ITS, nad6 and 28S in the present study, ITS showed the highest species resolution because it could resolve the pairs *M.whitei*/*kriegeriana* and *M.blechni*/*woodwardiana*.

The alternative barcode nad6 has been tested on different rust species (Vialle et al. 2009, Feau et al. 2011, Vialle et al. 2013). It was chosen for the present study because, in a study on *Melampsora* spp., it was the only barcode amongst six tested barcodes (ITS, 28S; CO1, nad6, MS277, MS208) that could distinguish the species *Melampsoralaricis-tremulae* and *Melampsorapinitorqua* (Vialle et al. 2013) on the basis of a Single Nucleotide Polymorphism. As seen from the branch lengths in Figure 1 and Figure 2 (compare also the scale bars), nad6 sequences show the lowest variation between the sections *Milesina*, *Scolopendriorum*, and *Vogesiacae* as compared to the other two markers. Within the sections *Milesina* and *Scolopendriorum*, the sequences were completely identical amongst the species. This low variation makes this marker more suitable for studies on infrafamiliar level than for species distinction on the infrageneric level.

The fungal barcode 28S rDNA is the second most widely used, following ITS (Schoch et al. 2012). It it used for phylogenetic analysis of rusts on the infrafamiliar or higher taxonomic level (Maier et al. 2003, Aime et al. 2006, Yun et al. 2011), but also the identification of closely related species (V, Beenken 2014, Maier et al. 2016, Beenken et al. 2017, Demers et al. 2017, Zhao et al. 2017). In the studies that directly compare ITS with 28S data, species resolution of ITS and 28S are comparable (Ali et al 2016, Beenken et al. 2012, Beenken et al. 2017, Feau et al. 2011) or ITS (namely the sub region ITS2) shows a slightly better resolution (Kenaley et al 2018, McTaggart and Aime 2018). However, when species could not be distinguished with ITS data, distinction was also not possible with 28S data. For instance, European specimens of *Coleosporium* species *C.euphrasiae* (Schumach.) G. Winter, *C.campanulae* (Pers.) Lév. and *C.senecionis* (Pers.) Fr., each occurring on telial hosts in different plant families, could neither be distinguished by ITS nor by 28S sequences. This is also clearly the case in the present study for the *Milesina* species in the sections *Milesina* and *Scolopendriorum* that colonise different fern species, but have almost identical ITS and 28S sequences.

One possible solution to lacking species resolution is to declare all specimens with the same ITS sequence data as one species, which was the original concept of ITS barcoding (Schoch et al. 2012). However, it is not only the telial host that differs between the Milesina species in the section Scolopendriorum, but also the features of the urediniospores (see identification key and Table 4). In the case of contradicting results, it is not advisable to weight the ITS data as more reliable than the morphological/host data. In the ascomycete genus *Fusarium*, several species complexes could not be resolved by ITS data, but with newly developed barcodes TOPI (Topoisomerase I) and PGK (phosphoglycerate kinase, Al-Hatmi et al. 2016). It is also possible that, for the rust fungi, new barcodes can be developed on the basis of genomic data (Aime et al. 2018b) that finally allow morphologically determined species to be resolved.

### Success of sequencing

Not all specimens studied morphologically were used for sequencing (i.e. old specimens, type specimens, specimens with little spore material) and not all of those specimens, where DNA was extracted, were successfully sequenced. The rate of successful ITS sequencing (63%) is relatively low. In a previous study on *Melampsora* rust fungi on *Salix*, the rate of ITS sequencing success (93%) was much higher (Bubner et al. 2014). The *Melampsora* study comprised exclusively freshly collected specimens not older than half a year, whereas we also analysed herbarium material that was several years old. However, freshly collected specimens (from 2017) also failed, while the two oldest successful specimens are from 1999. Therefore, it is not only the age of the samples that explains the low success of sequencing. It is possible that the small and fragile sori of *Milesina* species (in comparison, for instance, to *Melampsora* sori on *Salix*) are more prone to DNA decay on the herbarium specimens.

Even more surprising is the low success rate for the 28S sequencing. The 28S sequencing was performed only on samples with successful ITS sequencing. Template DNA should be present because both loci belong to the same multicopy rDNA region on the nuclear DNA. Despite this linkage, 28S is reported to have a PCR success rate of only 80% as compared to ITS in a large scale study on Basidiomycota including Pucciniomycotina (Schoch et al. 2012). Nevertheless, in recent phylogenetic studies on rusts, often concatenated alignments of ITS and 28S are used (e.g. Beenken 2017, Demers et al. 2017, McTaggart and Aime 2018, Vialle et al. 2013) which requires that both ITS and 28S can be sequenced. Although phylogenetic studies rarely report a success rate, it can be assumed that routine sequencing of both loci is possible. The low sequencing success in *Milesina* could be a genus-specific problem. We used 28S primers (ITS4BRF, LR5) which Vialle et al. (2013) successfully used for sequencing *Melampsora* spp. on poplars for *Milesina* spp., however, with much less success. Possibly 28S rDNA of *Milesina* is more difficult to sequence than in other rust genera. Other primer combinations for sequencing 28S rDNA are available and could be tested. The requirements of further testing demonstrate that, in the genus *Milesina*, species identification by barcode sequencing is still far from being routine.

### Section Vogesiacae

The support values for section Vogesiacae are smaller than for the other three *Milesina* sections when *M.exigua* is included. Interestingly, the support values are 100/1/100 for an ITS tree with *Pucciniagraminis* as outgroup. The decision to place both *M.vogesiaca* and *M.exigua* into one section is more strongly supported by morphological than by molecular data. *Milesinavogesiaca* and *M.exigua* are the only two species that have urediniospores without ornamentation and a bizonate position of germ pores. Further support for a molecularly and morphologically defined clade is given through *Uredinopsisfilicina*. In the ITS phylogram, it groups behind a node with high support values that includes both *M.vogesiaca* and *M.exigua*. The inclusion of *U.filicina* within a clade, that comprises all *Milesina* species (also *M.carpatica*), indicates that the genus *Milesina* is paraphyletic. The paraphyly is also indicated in the nad6 and 28S phylograms. Furthermore, *M.vogesiaca*, *M.exigua* and *U.filicina* form a group with high support values in 28S phylogram.

By morphology of urediniospores, *U.filicina* (the type species of *Uredinopsis*) is similar to the two Milesina species in the section Vogesiacae because it also has smooth urediniospores (Gäumann 1959). Germ pores have not been analysed so far. Germ pores are reported for two North American species, *U.osmundae* Magnus and *U.atkinsonii* Magnus (Moss 1926). Urediniospores are smooth and show the same bizonate position of germ pores as documented for *M.vogesiaca* and *M.exigua*. A recent study on molecular age estimates in Pucciniales presents 28S data of Melampsorineae that also comprises a limited selection of *Uredinopsis* and *Milesina* specimens (Figure 3 in Aime et al. 2018a). The species *U.osmundae*, *U.filicina* and *M.vogesiaca* group together, while *M.scolopendrii* and an undetermined *Milesina* species form a sister clade. This confirms the topology of the trees in the present study. The molecular and the morphological data indicate that at least *U.filicina* is actually a *Milesina* species in the clade *Vogesiaceae*. To place *U.filicina* (and possible other species of *Uredinopis*) in the genus *Milesina*, however, requires a more comprehensive sampling of *Uredinopsis* species and sequencing of both ITS and 28S rDNA (study in preparation).

### Host alternation in section Milesina

Amongst the 11 sequences of specimens found on the aecial host *Abiesalba*, nine could be assigned to the section Milesina. Although this section contains four species, the question which species is able to form aeciospores on *Abiesalba* can be narrowed down to three species. Only telial hosts of *M.blechni*, *M.kriegeriana* and *M.whitei* grow in the distribution area of *Abiesalba* in Europe. Therefore, *M.woodwardiana* can be excluded, because the host *Woodwardiaradicans* is restricted to Macaronesia and the Mediterranean (Hohenester and Welss 1993, Li et al. 2016) from where no *Milesina* sequences from the aecial host (*Abies* spp.) are available. In addition, *M.woodwardiana* obviously does not form telia and, consequently, no basidia and basidiospores. Basidiospores, however, are necessary to infect *Abies*.

The answer to the question which of the two species *M.whitei* / *M.kriegeriana* has an alternate host needs further field observation and experimental studies (inoculation experiments). Our specimens most probably belong to *M.kriegeriana*, because they were all collected in the Black Forest area (SW Germany) where we found *M.kriegeriana* many times on the telial host but not *M.carpatica*. Inoculation experiments should not only include the hosts of *M.whitei* (*Polystichum* spp.) and *M.kriegeriana* (*Dryopteris* spp.), but also *Struthiopterisspicant*, the host of *M.blechni*. This would further help to answer the question whether *M.blechni* and *M.kriegeriana* are distinct species or not. Despite the SNP at position 381, both species are very similar in urediniospore morphology. Inoculation experiments would provide further arguments to clarify the status of the two species. Another approach to analyse both host alternation and species distinction in the section Milesina would be to measure gene flow between the aecial (*Abies*) and the different telial hosts (ferns) by population genetics. Gene flow measurements in rust fungi have been applied to *Melampsoralarici-populina* (Barres et al. 2012; Elefsen et al. 2014).

## Conclusion

Both morphological features of the urediniospores and ITS sequences provide data to distinguish subgeneric groups (sections) in the genus *Milesina*. Apart from the two related species, *M.blechni* and *M.kriegeriana* in the section Milesina, morphological characteristics of urediniospores are sufficient to distinguish all European species in the genus *Milesina*. In contrast, ITS, nad6 and 28S barcodes worked only for the sections *Carpaticae* and *Vogesiacae* and failed to resolve species in the sections *Milesina* and *Scolopendriorum*. Therefore, morphology of urediniospores, in conjunction with host determination, is still a more secure and faster tool to identify species in *Milesina* on the telial host. Other markers have to be developed for quicker and more secure identification with barcodes.

## Supplementary Material

XML Treatment for Milesina blechni

XML Treatment for Milesina carpatica

XML Treatment for Milesina exigua

XML Treatment for Milesina feurichii

XML Treatment for Milesina kriegeriana

XML Treatment for Milesina magnusiana

XML Treatment for Milesina murariae

XML Treatment for Milesina polypodii

XML Treatment for Milesina scolopendrii

XML Treatment for Milesina vogesiaca

XML Treatment for Milesina whitei

XML Treatment for Milesina woodwardiana

XML Treatment for Milesina

XML Treatment for Milesina sect. Milesina

XML Treatment for Milesina sect. Vogesiacae

XML Treatment for Milesina sect. Scolopendriorum

XML Treatment for Milesina sect. Carpaticae

## Supplementary materials

Supplementary material 1 *Milesina* ITS Data type: multimedia Explanation note: Fig. S1. This is an open access article distributed under the terms of the Creative Commons Attribution License (CC BY 4.0), which permits unrestricted use, distribution, and reproduction in any medium, provided the original author and source are credited.Ben Bubner, Ramona Buchheit, Frank Friedrich, Volker Kummer, Markus Scholler

Supplementary material 2 *Milesina* Nad6 Data type: multimedia Explanation note: Fig. S2. This is an open access article distributed under the terms of the Creative Commons Attribution License (CC BY 4.0), which permits unrestricted use, distribution, and reproduction in any medium, provided the original author and source are credited.Ben Bubner, Ramona Buchheit, Frank Friedrich, Volker Kummer, Markus Scholler

Supplementary material 3 *Milesina* 28S Data type: multimedia Explanation note: Fig. S2. This is an open access article distributed under the terms of the Creative Commons Attribution License (CC BY 4.0), which permits unrestricted use, distribution, and reproduction in any medium, provided the original author and source are credited.Ben Bubner, Ramona Buchheit, Frank Friedrich, Volker Kummer, Markus Scholler
